# Yap- and Cdc42-Dependent Nephrogenesis and Morphogenesis during Mouse Kidney Development

**DOI:** 10.1371/journal.pgen.1003380

**Published:** 2013-03-21

**Authors:** Antoine Reginensi, Rizaldy P. Scott, Alex Gregorieff, Mazdak Bagherie-Lachidan, Chaeuk Chung, Dae-Sik Lim, Tony Pawson, Jeff Wrana, Helen McNeill

**Affiliations:** 1 Samuel Lunenfeld Research Institute, Mount Sinai Hospital, Toronto, Ontario, Canada; 2 Department of Molecular Genetics, University of Toronto, Toronto, Ontario, Canada; 3 Korea Advanced Institute of Science and Technology, Daejeon, Korea; University of Michigan, United States of America

## Abstract

Yap is a transcriptional co-activator that regulates cell proliferation and apoptosis downstream of the Hippo kinase pathway. We investigated *Yap* function during mouse kidney development using a conditional knockout strategy that specifically inactivated Yap within the nephrogenic lineage. We found that Yap is essential for nephron induction and morphogenesis, surprisingly, in a manner independent of regulation of cell proliferation and apoptosis. We used microarray analysis to identify a suite of novel Yap-dependent genes that function during nephron formation and have been implicated in morphogenesis. Previous *in vitro* studies have indicated that Yap can respond to mechanical stresses in cultured cells downstream of the small GTPases RhoA. We find that tissue-specific inactivation of the Rho GTPase *Cdc42* causes a severe defect in nephrogenesis that strikingly phenocopies loss of *Yap*. Ablation of *Cdc42* decreases nuclear localization of Yap, leading to a reduction of Yap-dependent gene expression. We propose that Yap responds to Cdc42-dependent signals in nephron progenitor cells to activate a genetic program required to shape the functioning nephron.

## Author Summary

The mammalian kidney undergoes reiterative and stereotypical morphogenetic changes to create the elaborately convoluted adult nephron, the functional filtration unit of the kidney. How these sequential morphological events are controlled remains poorly understood. Here we show that the transcriptional activator Yap is essential in the developing murine kidney. *Yap* mutants have reduced nephrogenesis and defective morphogenesis. Yap function in nephrogenesis is independent of its previously described role in regulation of cell proliferation and apoptosis. Instead, Yap activity is needed for proper expression of a suite of genes that control cell signaling and cell structure. Remarkably, we find that ablation of *Cdc42* phenocopies loss of *Yap*. We show that Cdc42 is essential for nuclear access of Yap, both *in vivo* and in tissue culture studies. Taken together, our work shows that *Yap* and *Cdc42* are essential for the cell fate and morphogenesis decisions necessary to shape functioning nephrons, and suggests that *Yap* functions downstream of *Cdc42* during kidney development.

## Introduction

Nephrons are the functional units of the kidney. Variability in nephron number (300,000 to 1 million in each kidney [1]) in human depends on both environmental and genetic factors. Low nephron number at birth correlates with increased incidence of renal failure later in life [2]. Thus, it is critical to understand the molecular mechanisms underlying nephron induction and patterning.

**Figure 1:**
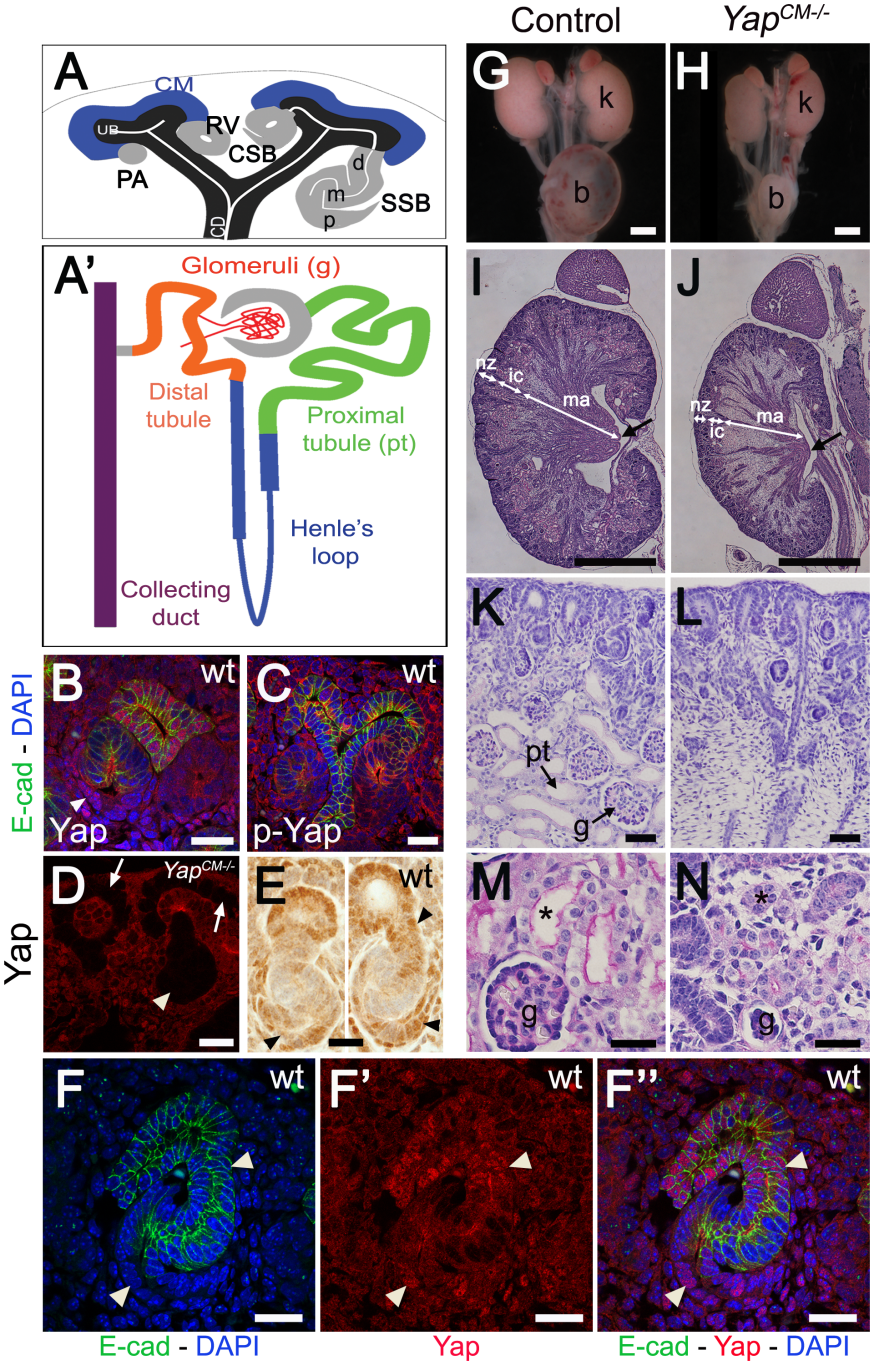
*Yap* is required for kidney development. (A) Stages of nephrogenesis and their relationship to the UB (black) tips. Signals released from UB tips induce mesenchyme cells to condense around UB tips forming the CM (blue). Some of these CM cells aggregate forming the PA that converts into epithelial RV. The late RV fuses with UB tips and develops into comma (CSB) and S-shaped (SSB) body. (A′) Schematic diagram of the nephron components. (B) Confocal images for Yap, E-cadherin and DAPI staining in late RV at E14.5. Nuclear Yap is observed in the proximal segment of the RV (arrowheads), while Yap expression disappears in Six2:Cre expressing cells (D - arrows point to CM cells, arrowhead points to an early nephron). (C) Confocal images of p-Yap/E-cadherin/DAPI staining shows ubiquitous p-Yap expression. Individual channels images are in Figure S2. (E) Immunohistochemistry using Yap/Taz antibody in RV and SSB shows a similar expression pattern observed with Yap antibody in previous panels (arrowheads). (F–F″) Confocal images for Yap/E-cadherin/DAPI staining in SSB at E14.5. Nuclear Yap is observed in proximal and distal segments of the SSB (arrowheads). (G,H) Macroscopic view of the urogenital system from wild-type and *Yap* mutant kidneys at P0. Note bilateral reduction in kidney size of mutant compared to control and empty bladder in mutant animals. (I,J) PAS staining of P0 kidneys from wild-type and *Yap^CM−/−^* animals. Arrows point to the papilla. (K,L) Closer view of the cortical zone shows limited nephrogenesis in *Yap^CM−/−^*. (M,N) Higher magnification shows abnormal glomeruli structure and tubules with barely discernable lumens (asterisk) in *Yap^CM−/−^*. k: kidney; b: bladder; cd: collectiong duct; csb: comma-shaped body; d: distal; g: glomeruli; ic: inner cortex; ma: medulla; m: medial; nz: nephrogenic zone; p: proximal; pt: proximal tubule; ssb: S-shaped body. Scale bars represent 25 µm (B–F″; M–N), 1 mm (G–J), 200 µm (K,L).

Kidney organogenesis is a remarkably orchestrated, reiterated process that depends on reciprocal signaling between the epithelial ureteric bud (UB) and the surrounding metanephric mesenchyme [3]–[6]. Signaling from the mesenchyme induces successive rounds of UB branching, generating the collecting duct system of the kidney. Surrounding the UB are self-renewing mesenchymal progenitor cells called the cap mesenchyme (CM). A subset of CM cells is reciprocally induced by the UB to form a pretubular aggregate (PA), which subsequently undergoes a mesenchyme-to-epithelial transition (MET) to form a renal vesicle (RV). The RV then undergoes morphogenesis, first changing into a comma-shaped body (CSB) that then elongates and folds back on itself to form a S-shaped body (SSB) (Figure 1A). Finally, the SSB further elongates and undergoes patterned differentiation to give rise to the various segments of the nephron (Figure 1A′). This process is repeated thousands of times, resulting in the stereotypical structure of the mature nephron which includes the distal tubules, proximal tubules, Henle's loops and glomeruli. How this intricate morphogenetic process is regulated is not fully understood.

The Hippo pathway is a highly conserved kinase cassette that regulates tissue growth in metazoans by controlling the activity of Yap and Taz (reviewed in [7]–[9]). Yap and Taz are closely related transcriptional co-activators that control expression of genes that promote cell proliferation and inhibit apoptosis. When the Hippo kinases Mst and Lats are active, Yap and Taz are phosphorylated and excluded from the nucleus. Loss of Hippo signaling leads to unrestricted proliferation in flies and mammals, and has been linked to a variety of cancers (reviewed in [10], [11]). *Yap* knockout (*Yap^−/−^*) embryos die at embryonic day 8.5 (E8.5) [12], and *Taz^−/−^* mice have polycystic kidney disease [13], [14]. *Yap^−/−^;Taz^−/−^* embryos die prior to the morula stage with defects in trophectoderm specification, indicating redundant roles in early embryonic development [15].

Blocking the inhibitory effects of Mst/Lats signaling on Yap, either through disruption of Salvador (*Sav1/WW45*) or by forced expression of a constitutively active form of Yap, leads to hyperproliferation of cells in the gut and skin [16], [17]. Hippo signaling has also been shown to restrict heart size in mice [18]. Upstream of Hippo kinases lie a number of cell surface regulators, which include cadherins, cell polarity complexes and GPCRs [19]. These and other data in flies, fish and mice (reviewed in [7], [8], [20]) have led to a model in which Yap and Taz primarily function to regulate tissue growth.

Surprisingly, recent studies in tissue culture demonstrate that Yap and Taz also respond to mechanical stresses [21], [22]. Plating cells on a rigid substrate induces the nuclear localization of Yap and Taz, promoting transcription of Yap/Taz targets that enhance cell proliferation and inhibit apoptosis. Disruption of the cytoskeleton has also been shown to regulate cell proliferation and Hippo pathway activity in *Drosophila* (reviewed in [20]). These studies specifically highlight the importance of the establishment of cytoskeletal tension for Yap/Taz dependent-mechanotransduction and implicated RhoA activity and the integrity of stress fibers in mediating Yap/Taz responses to mechanical cues. The *in vivo* developmental relevance of these findings has not been addressed before. In addition, no study has examined to date whether the activity of other Rho GTPases can influence Yap function.

We have investigated the role of Yap signaling in the context of nephrogenesis in the murine kidney. To bypass the early lethality of *Yap* ^−/−^ mutants, we used tissue-specific deletions to study Yap function during nephron formation. We found that *Yap* conditional mutants display early defects in nephron induction, and that the stereotypical morphogenesis that remodels the RV into SSB is disrupted. Importantly, these early defects occur independently of major alterations in proliferation or apoptosis. Using microarrays, we identified a suite of genes whose expression depends on Yap during kidney morphogenesis. Intriguingly, we find that loss of the small Rho GTPase *Cdc42* leads to a reduction of nuclear Yap in the CM and cytoplasmic accumulation of Yap in cultured fibroblasts. Importantly, nephrogenic inactivation of *Cdc42* leads to loss of Yap-dependent gene expression. Moreover, *Cdc42* and *Yap* removal in the CM leads to remarkably similar morphological defects and abnormalities in nephron gene expression. Together these data support a model in which *Cdc42* acts upstream of *Yap* in nephron progenitor cells, to promote gene expression required to establish and shape nephrons.

## Results

### Deletion of *Yap* in the CM results in abnormal nephron formation

To investigate a potential function of *Yap* in nephrogenesis, we first stained developing kidneys with antibodies to Yap, and found that Yap was dynamically expressed throughout nephrogenesis. As a transcriptional co-activator the function of Yap is primarily regulated at the level of access to the nucleus [23]. Yap is expressed in the ureteric compartment and cortical stromal cells, with lower levels of expression in the CM (Figure 1B, 1E). Strikingly, we noted that the distribution of Yap is regulated spatially and temporally during nephrogenesis. In early nephrogenic structures, Yap is strongly expressed in proximal cells of the RV (Figure 1B, 1E) and in most distal and proximal cells of the SSB (Figure 1E, 1F–1F″). This dynamic expression pattern was seen using two different Yap antibodies, and was lost upon deletion of *Yap* from the CM using Six2:Cre (Figure 1D and Figure S1).

Yap localization in the nucleus is often regulated by phosphorylation. We stained embryonic kidneys with antibodies that recognize Yap phosphorylated at S127, a site that is phosphorylated by Lats in response to Hippo activation [24], and found that phospho-Yap staining is detectable throughout kidney development (Figure 1C and Figure S2). However, we found no correlation between phospho-Yap staining and Yap localization in the RV or SSB stages.

To directly assess the function of *Yap* during nephron formation, we removed *Yap* from the CM with *Six2:Cre^TGC/+^* [25]. Since all components of the nephron, from the glomerulus to the distal tubule derive from Six2-expressing CM cells, this system removes *Yap* from the CM and all of its epithelial derivatives (i.e. podocyte, Bowman's capsule, proximal tubule, Henle's Loop and distal tubule). We found that *Six2:Cre^TGC/+^ Yap^flox/flox^* (termed *Yap^CM−/−^*) newborns were obtained at Mendelian ratios. However, despite successful feeding, *Yap^CM−/−^* animals died within 48 hours of birth. Gross anatomical examination revealed that neonatal (P0) *Yap^CM−/−^* animals had hypoplastic kidneys and an empty bladder suggesting a failure to produce urine (Figure 1G, 1H). Histological examination of E18.5 kidney sections revealed a smaller papilla and a reduced nephrogenic zone. Convoluted renal tubules and glomeruli were not distinguishable in the inner cortex of the mutant, and the medulla was mainly composed of collecting ducts, suggesting a dramatic reduction in Henle's loop formation (Figure 1I–1L). *Yap^CM−/−^* mutant kidneys had few detectable glomeruli and proximal tubules (Figure 1K–1N). Strikingly, the rare glomeruli observed in *Yap^CM−/−^* mutants were ultrastructurally abnormal characterized by simplified capillary tufts ensheathed with podocytes having effaced foot processes (Figure S3).

**Figure 2:**
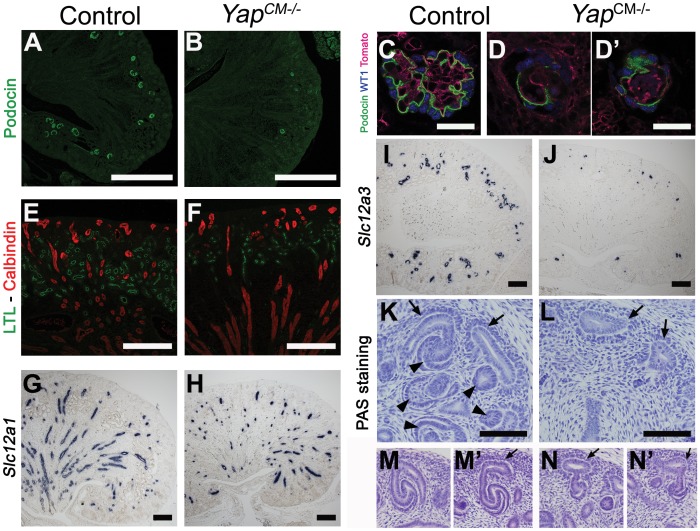
Loss of CM-derived epithelial structures and abnormal morphogenesis in *Yap* mutants. (A–J) Sections of P0 kidneys stained using late nephron markers confirm abnormal nephron formation in *Yap^CM−/−^* kidneys. Glomeruli (Podocin, A,B; Podocin-WT1-Tomato lectin, C–D′). Proximal tubules (LTL, E,F). Henle's loop (*Slc12a1*, G,H). Distal tubules (*Slc12a3*, I, J). (K,L) Overview of an E14.5 nephrogenic zone reveals the presence of CM cells (arrows) in both genotypes, but CM-derived epithelial structures (arrowheads) are greatly reduced in mutant when compared to control littermates. (M–N′) Higher magnification shows histological morphology defects of mutant SSB compared to wild-type controls at E13.5. Scale bars represent 500 µm (A,B), 50 µm (C–D′), 200 µm (E–J), 100 µm (K–L).

Labeling with *Dolichos Biflorus* Agglutinin (DBA) lectin or Calbindin (Figure S4 and data not shown) confirmed that a branched collecting duct system was present as expected, as this structure does not derive from the CM. To determine which nephron compartment was affected by *Yap* inactivation, we used markers of distinct CM-derived nephron segments. Podocin staining labeled numerous glomeruli in wild-type kidneys, however, considerably fewer podocin-positive structures were detected in *Yap^CM−/−^* at birth, consistent with the reduced number of glomeruli seen in histological analysis (glomeruli number per section at P0: control:50±5; *Yap^CM−/−^*:6±2; ***p<0.001. Figure 2A, 2B). Furthermore, the few glomeruli observed in *Yap^CM−/−^* mice had abnormal structures as seen by triple staining with podocin, WT1 and tomato-lectin (Figure 2C–2D′ and Figure S3). Yap and phospho-Yap antibodies failed to stain the Six2-positive compartments in *Yap^CM−/−^* (Figure 1D, low and high magnification image panels are shown in Figure S1 and S2), suggesting that *Yap* excision was efficient, and that the rare nephron derivatives that form in mutants are likely not due to incomplete inactivation of *Yap*. Examination of markers at E18.5 revealed a dramatic loss of *Lotus tetragonolobus* lectin (LTL)-positive proximal tubule structures (Figure 2E, 2F). Interestingly, the morphogenesis of the remaining LTL-positive tubules was severely affected as they have barely discernable lumens at E18.5 (Figure 1M, 1N and Figure 2E, 2F). Staining for Ezrin, LTL and Par3 was normal in the residual tubules, indicating that cell polarity was retained (Figure S5). The reduced lumens may reflect an absence of filtration due to the dramatically reduced number of glomeruli. Strikingly, *Yap^CM−/−^* kidneys also have defects in Henle's loop (*Slc12a1*) and distal tubule (*Slc12a3*) formation (Figure 2G–2J). Thus Yap is necessary in CM cells for normal nephron development.

The CM plays an essential role in supporting branching morphogenesis of the developing kidney. To determine if loss of *Yap* from the CM alters branching, we analyzed the number of ureteric tips at different time-points using immunofluorescent staining with antibodies to Calbindin, which marks both the CD and the UB tips (Figure S4). While similar branching is observed in wild-type and *Yap^CM−/−^* kidneys at E14.5, the number of UB tips slightly decreases at E16.5 in *Yap* mutants with a significant reduction of tip number at P0. Thus loss of *Yap* in the CM does not affect early branching but has late-onset impairment of branching.

### *Yap* deletion impairs nephrogenesis and S-shaped bodies' morphogenesis

**Figure 3:**
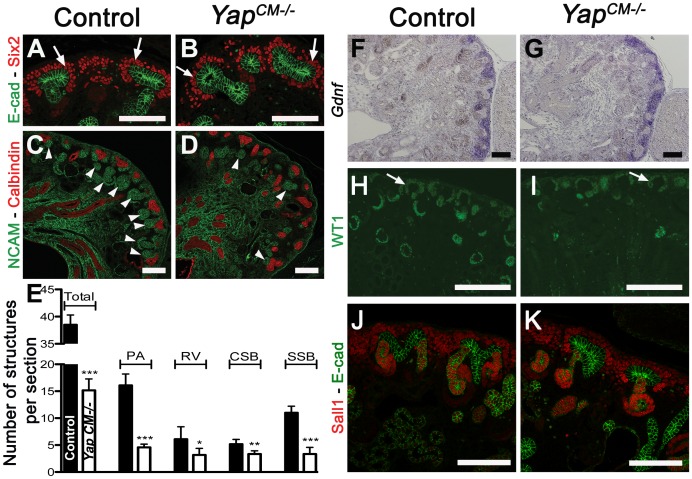
*Yap* deletion impairs nephron induction, without affecting self-renewal of the CM population. (A,B) Immunostaining analysis for Six2 (E14.5) shows no change in expression pattern in both genotypes (arrows). E-cadherin was used to visualize the UB compartment. (C,D) Dramatic reduction in nephrogenesis visualized by loss of NCAM-expressing structures (arrowheads) in the nephrogenic zone of *Yap* mutant compared to wild-type (E16.5). Note the reduced NCAM expression in CM cells. Calbindin highlights the UB and CD. (E) Quantification of early nephron structures in E15.5 controls (black columns) and *Yap* mutants (white columns) based on NCAM staining. Total***: p<0.0001; PA***: p<0.0001; RV*: p = 0.0209; CSB*: p = 0.0018; SSB***: p<0.0001. (F,G) ISH analysis shows maintained *Gdnf* expression in CM of control and *Yap* mutants (E15.5). (H,I) WT1 staining (E18.5) reveals staining in CM cells (arrows) for both genotypes, and dramatic reduction in number of renal MET-derived structures in mutants compared to wild-type. (J,K) Immunostaining analysis for the CM marker Sall1 (E14.5) shows no change in expression pattern in both genotypes. E-cadherin was used to visualize the UB compartment. Scale bars represent 100 µm.

**Figure 9:**
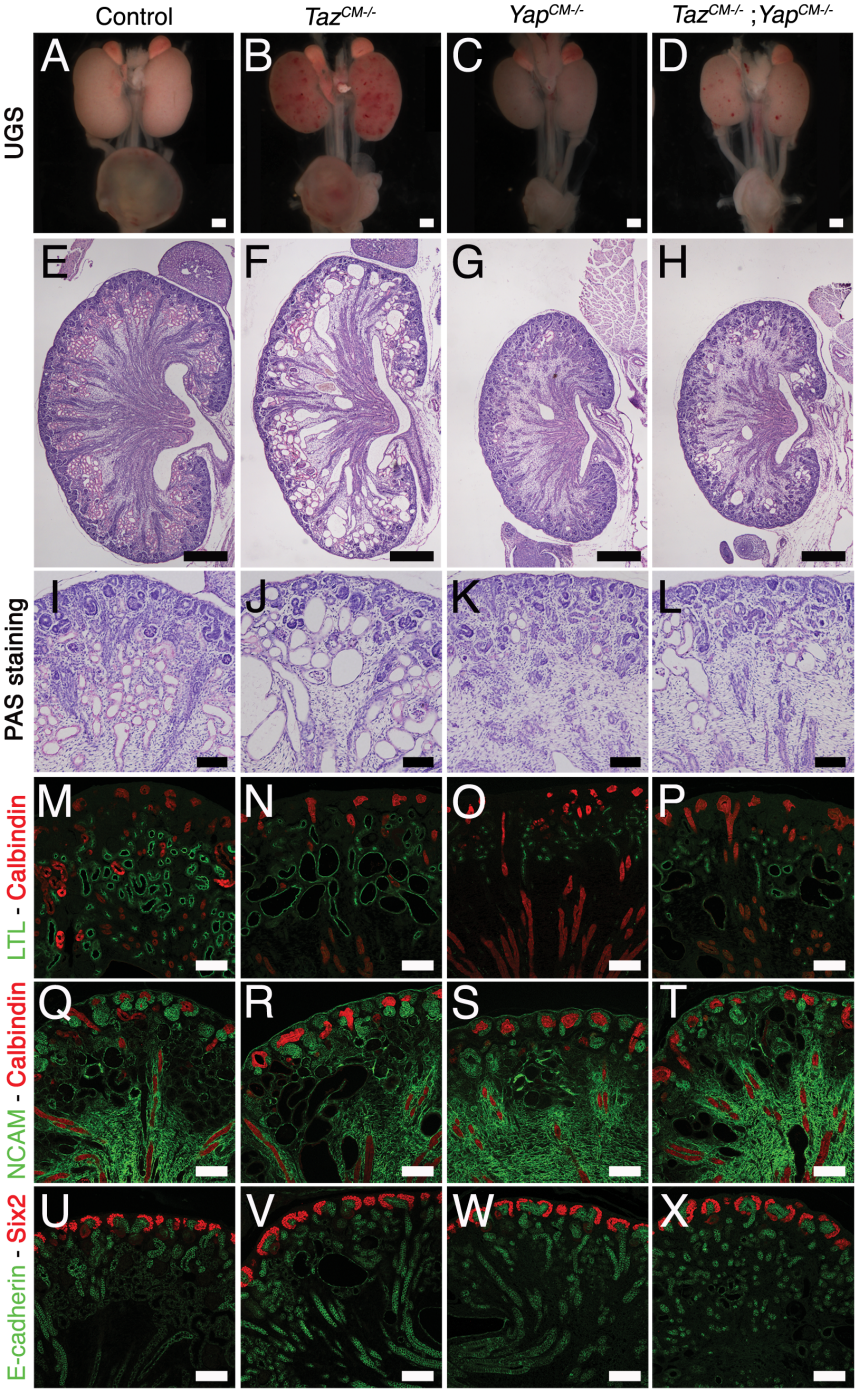
*Yap* and *Taz* have distinct roles during nephrogenesis. (A–D) Macroscopic view of the urogenital system from wild-type, *Taz^CM−/−^*, *Yap^CM−/−^* and *Taz^CM−/−^;Yap^CM−/−^* double mutant kidneys at P0. (E–H) PAS staining of P0 kidneys. (I–L) Closer view of the cortical zones. (M–P) LTL staining for each genotype. (Q–T) NCAM staining for all genotypes. (U–X) Six2 staining reveals progenitor cell population in all genotypes. Scale bars represent 500 µm (A–H) and 100 µm (I–X).

To determine the developmental origin of the defective nephrogenesis in *Yap^CM−/−^*kidneys, we examined kidney development from E13.5 to E18.5. Nephrogenesis occurs in a repetitive manner, with new nephrons being formed throughout development at the outer cortex of the kidney. This process is highly regulated, involving both inductive and repressive signals (reviewed in [4]). Six2-expressing precursor cells residing in the cortex self-renew to replenish a pool of mesenchymal cells that are then transformed into nascent nephrons [25]. Maintenance of the progenitor population requires Six2, as deletion of *Six2* results in premature differentiation of the CM cells [23]. Histological analysis revealed that *Yap^CM−/−^* kidneys have limited nephrogenesis (Figure 2K, 2L) with abnormal morphogenesis of SSB (Figure 2M–2N′). CM cells in both *Yap^CM−/−^* and wild-type kidneys were detected by histological analysis at E14.5 (Figure 2K–2N′) and by Six2, *Gdnf* and Sall1 expression (Figure 3A, 3B, 3F–3K), indicating that nephrogenic precursors cells are present in *Yap* mutant kidneys. Clear Six2 staining is obvious even at P0 (Figure 9W), although there is a mild reduction in the total number of Six2-positive cells in *Yap* mutant kidneys compared to wild-type kidneys (Figure S6). In contrast, however, the number of nascent nephrons (PA, RV, CSB and SSB) was clearly and dramatically reduced in *Yap* mutant kidneys early in development, as revealed by histological analysis (Figure 2K, 2L), NCAM staining (Figure 3C, 3D) and WT1 staining (Figure 3H, 3I). Quantification of NCAM-positive nephrogenic structures at E15.5 further validated a significant decrease in total nephrogenesis due to *Yap* deletion (Figure 3E). Since no change in branching morphogenesis could be detected at this stage (Figure S4), the limited nephrogenesis in *Yap* mutants is not secondary to impaired ureteric branching. Few PA could be detected in *Yap* mutant kidneys (Figure 3E), further showing that nephron induction is severely disrupted. In addition, the number of CM derived structures that reached the SSB stage in the mutant was dramatically reduced when compared to controls (Figure 3E). Thus, while the self-renewing capacity of CM cells is largely *Yap* independent, *Yap*-depleted CM cells are less potent to undergo nephrogenesis, and are unable to execute regulated morphogenesis to form regular SSB.

### Segmentation of the renal vesicle is independent of *Yap* function

**Figure 4:**
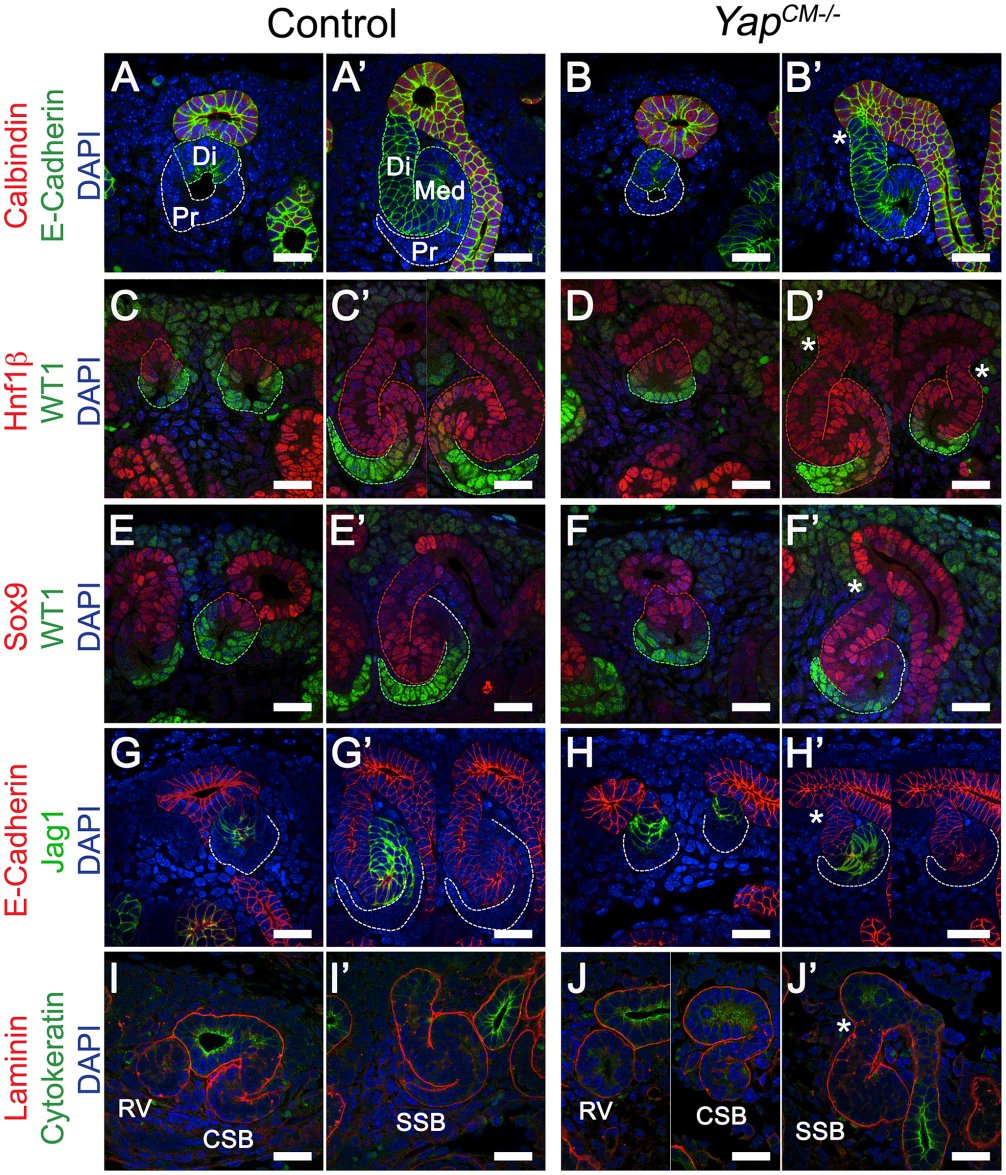
Characterization of segmentation in *Yap* mutant nephrons. (A–B′) Double staining for E-cadherin and Calbindin in RV and SSB. Co-staining for Hnf1ß/WT1 (C–D′) and Sox9/WT1 (E–F′) reveals normal segmentation of the RV with both proximal and distal segments. Similarly, SSB show normal segmentation. Note the reduced size of the proximal domain in *Yap*-null SSB (compare WT1 positive segment in *Yap* mutants (D′, F′) to controls (C′, E′). This is also apparent in B′ and J′. (G–H′) Immunofluorescence for E-cadherin and Jag1 reveals no change in specification of the distal RV and the medial segment of the SSB in both genotypes. Note the aberrant morphology (asterisk) of the site where the connection occurred between the SSB and the UE (B′,D′,F′,H′ and J′). (I–J′) Immunofluorescence using antibodies to Cytokeratin (UE) and Laminin (BM) shows that fusion occurred before the comma-shaped stages. All staining performed at E15.5. CSB: comma-shaped body; RV: renal vesicle; SSB: S-shaped body. Scale bars represent 25 µm. DAPI was used to counterstain nuclei.

Formation of a functioning nephron requires polarization of the emerging epithelium along a proximodistal axis to specify diverse cell types. Polarization and segmentation is detectable as early as the RV stage. Segmentation becomes clearly apparent at the SSB stage with distal-, medial- and proximal-specific gene expression. We examined nephron segmentation at both the RV and SSB stages. *Yap* deletion did not impair RV polarization as proximal (WT1) and distal (E-cadherin, Hnf1ß, Sox9, Jag1) markers showed similar expression patterns (Figure 4A–4H). Similarly, in later nephrogenic structures, no segmentation defect could be seen in distal and medial SSB (Figure 4A–4H, Distal:E-cadherin, Hnf1ß, Sox9; Medial:Jag1, Hnf1ß, Sox9; Proximal:WT1 [26]–[28]). However, *Yap*-null SSB have a reduced WT1 positive proximal segment (in particular compare Figure 4C′ versus 4D′; 4E′ versus 4F′) consistent with defects in proximal fate seen in P0 kidneys.

**Figure 5:**
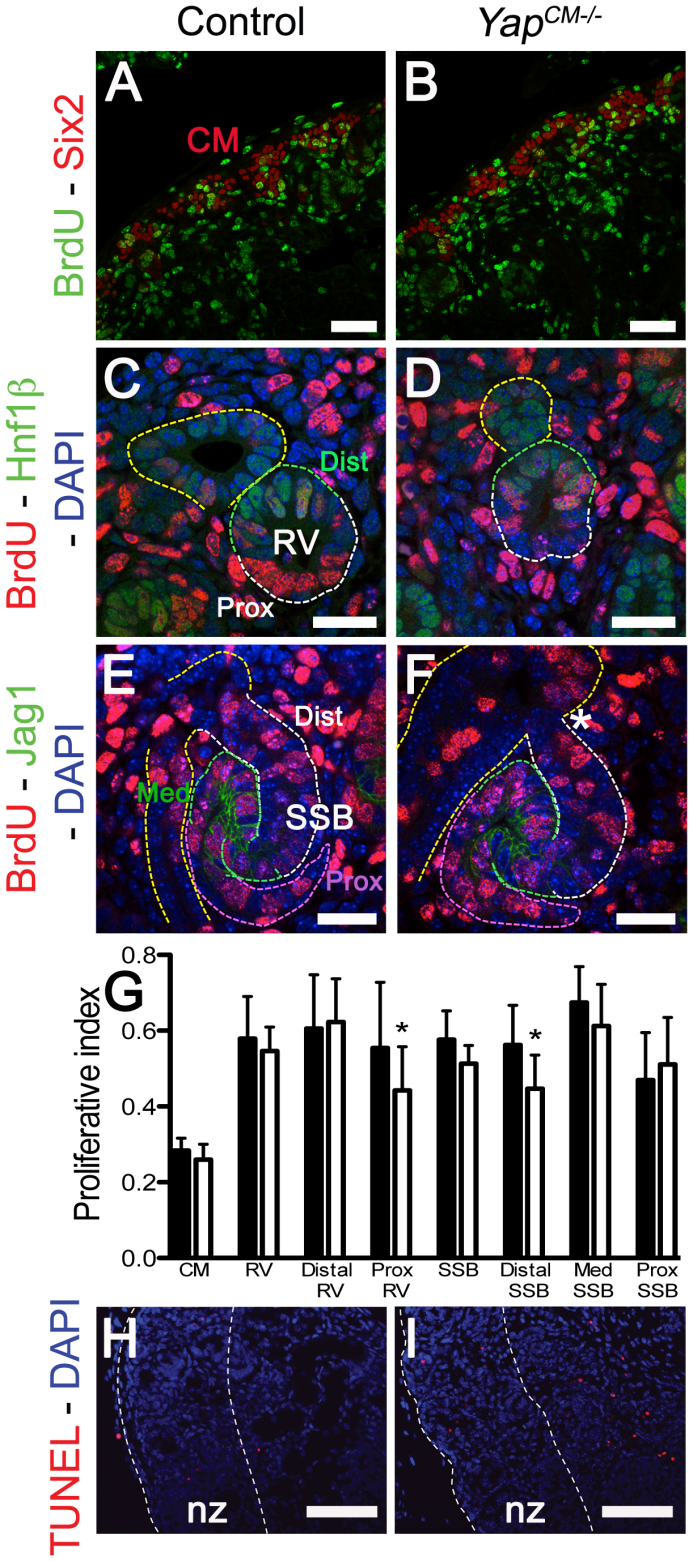
No major change in apoptosis or proliferation in *Yap* mutant kidneys. Confocal images of BrdU incorporation in condensing mesenchymal cells (A,B), renal vesicle (C,D) and SSB (E,F) at E15.5. (A,B) Co-staining with Six2 antibody was used to co-labeled cap mesenchyme cells. (C,D) Co-staining with Hnf1ß antibody was used to distinguish the distal (Dist) from the proximal (Prox) segment of the RV. (E,F) Jag1 antibody was used to identify the distal (Dist), medial (Med) and proximal (Prox) segments of the SSB. (G) Quantification of the proliferation index in controls (black columns) and *Yap* mutants (white columns) throughout nephrogenesis. Prox RV*: p = 0.0319; Distal SSB*: p = 0.0353. (H,I) TUNEL assay at E18.5 reveals no change in apoptosis in mutant nephrogenic zone (nz). There is often an increase in apoptosis in the later developing inner cortex in the *Yap* mutants. Scale bars represent 50 µm (A,B), 25 µm (C,F), 100 µm (H,I).

Formation of a functional nephron also requires fusion to the ureteric bud, a process that occurs at the late RV stage [29]. Staining with Laminin to mark the basement membrane (BM) and Cytokeratin to mark the ureteric epithelium (UE) of an early RV shows that the RV is surrounded by its own BM and separated from the adjacent UE by the ureteric epithelial BM in both controls and *Yap^CM−/−^* mutants (Figure 4I, 4J). At the comma stage (Figure 4I, 4J) fusion of the early nephron to the UE is complete in both genotypes as seen by a continuous BM. However, we note that *Yap^CM−/−^* mutants consistently display aberrant morphology at the connecting segment, where the SSB connects to the UE (asterisk, see also Figure 3K, Figure 5F, Figure S2B). In particular, the distal segment of the SSB does not correctly merge with the outermost edge of the UB.

### Loss of *Yap* does not significantly impact cell proliferation or apoptosis during early nephrogenesis

Yap, downstream of the Hippo pathway, has been extensively shown to regulate organ size by promoting cell proliferation and inhibiting apoptosis. We therefore analyzed cell proliferation throughout nephrogenesis to ascertain if altered proliferation or apoptosis could explain the morphological defects in *Yap* mutants. Quantification of BrdU incorporation in nephron progenitors cells (Six2 positive cells) did not reveal any significant changes in CM proliferation (n = 1,000 Six2 positive cells from 4 kidneys of each genotype, Figure 5A, 5B and 5G). Interestingly, while no significant changes could be detected in overall RV proliferation, or in distal RV proliferation, a slight reduction in proliferation was detectable in the proximal part of *Yap*-null RV (Hnf1ß negative, n = 16 RV per genotype - Figure 5C, 5D and 5G). Finally, we investigated proliferation in the distal (cells located between the UE and Jag1 expressing domain), medial (Jag1 positive cells) and proximal segments of the SSB (n = 12 SSB per genotype). Similarly to the RV stage, no significant change in proliferation could be detected in the overall SSB, however segment-specific analysis revealed slightly decreased proliferation in the distal segment of *Yap* mutant (Figure 5E, 5F and 5G). TUNEL staining in control and *Yap^CM−/−^* kidneys (E18.5) did not reveal any changes in apoptosis in mutants relative to controls (Figure 5H, 5I). Our data indicates that early defects in nephron formation in *Yap* mutants are not due to death of the nephrogenic cell population, nor to a dramatic failure to proliferate.

### No major disruptions of Notch or Wnt/ß-catenin signaling in *Yap^CM−/−^* kidneys

Recent studies have revealed functional interactions between Yap and ß-catenin, [18], [30]. While activation of the canonical ß-catenin signaling pathway is necessary for nephron formation, its repression is required for epithelialization to occur [31]. *Wnt9b* secreted from the UB induces mesenchymal condensation via canonical ß-catenin signaling, activating a molecular cascade involving *Fgf8*, *Wnt4*, *Pax8* and *Lim1* [32]. Expression of *Wnt9b* was unchanged in *Yap* mutant kidneys (Figure S7A, S7B). To see if ß-catenin signaling was affected in *Yap* mutant kidneys, we examined expression of ß-catenin signaling targets. Significantly, expression of the established Wnt target genes *Pla2g7*, *C1qdc2* and Lef1 were unchanged in *Yap^CM−/−^* kidneys (Figure S7C–S7H). Moreover, *Fgf8*, *Wnt4*, *Pax8*, and *Lim1* expression levels were also unchanged in *Yap^CM−/−^* kidneys (Figure S7I–S7P). Finally, removing one allele of *ß-catenin* in *Yap^CM−/−^* mice (by generating *Six2:Cre^TGC/+^ Yap^flox/flox^ ß-catenin(KO)^flox/+^* embryos) does not alter the *Yap^CM−/−^* phenotype (Figure S8). Taken together, these data indicate that Yap functions in nephron formation independently of major changes in the Wnt/ß-catenin signaling pathway.

The defects in glomeruli and proximal tubules that occur in *Yap^CM−/−^* kidneys are reminiscent of defects in Notch signaling [27]. We therefore assayed different members of the Notch pathway by *in situ* hybridization (ISH). In particular, no changes were detected in the expression levels of *Notch1*, *Notch2*, the ligand *Jagged1*, or the Notch targets *Hes1* or *Hes5* (Figure S7Q–S7X and data not shown). These data indicate that the loss of *Yap* does not lead to nephrogenesis defects via loss of Notch signaling.

### Whole-genome expression analysis identifies novel Yap-dependent genes in the kidney

**Figure 6:**
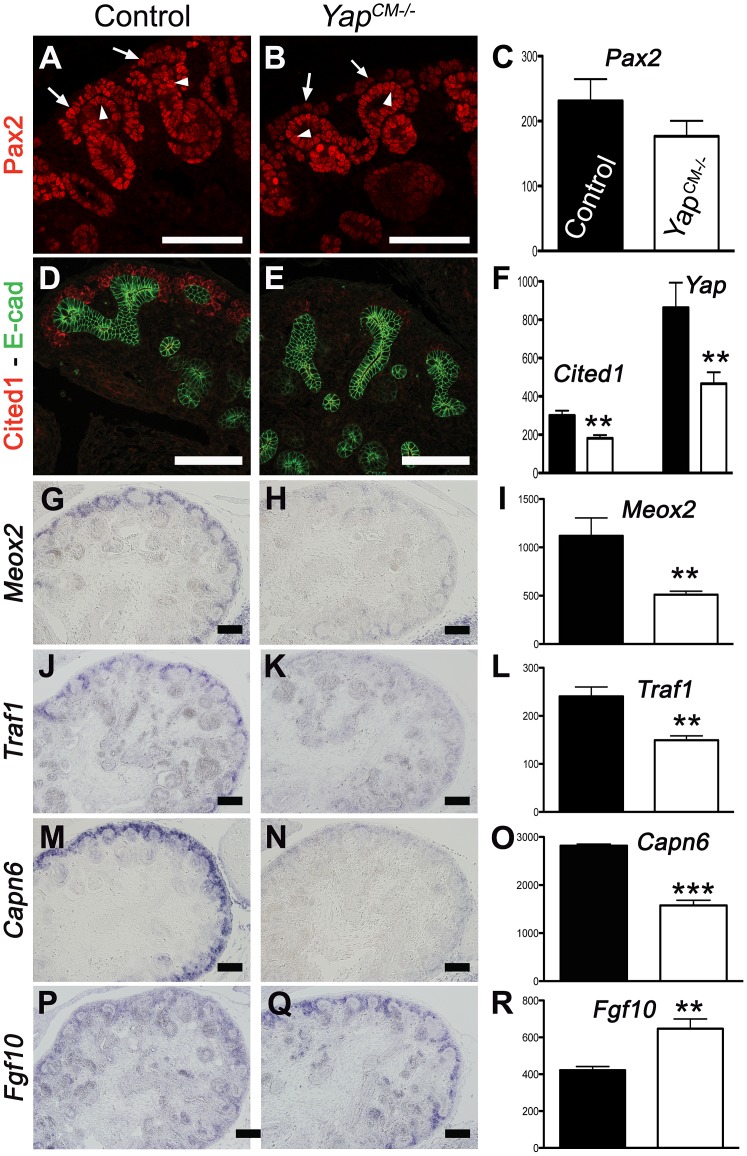
Transcriptional changes in *Yap* mutant CM progenitors cells. Expression of Pax2 (A), Cited1 (D), *Meox2* (G), *Traf1* (J) and *Capn6* (M) in control E14.5 kidneys, demonstrating expression in CM cells and other lineages. *Yap* deletion results in loss of gene expression of these genes in CM cells (B,E,H,K,N). Note the loss of Pax2 expression in the CM of *Yap* mutant (arrows in B) compared to control CM (arrows in A), while expression in the ureteric epithelium (arrowheads) remain unchanged. (P,Q) ISH reveals increase in levels of *Fgf10* expression specifically in nephron progenitor cells of *Yap* deficient kidneys compared to wild-type. (C,F,I,L,O,R) Graphical representation of the microarray data of control (black colums) and *Yap* mutant (white columns). (** p<0.001; *** p<0.0001). Scale bars represent 100 µm.

Since no defects were observed in ß-catenin or Notch signaling, and proliferation and apoptosis were largely unaffected, we sought an unbiased approach to determine the molecular basis of the defects seen in *Yap* mutants. We used whole-genome transcript profiling (Mouse Whole Genome-6 v2.0 BeadChip) at E13.75 to determine gene expression changes in *Yap* mutant kidneys. Of the ∼45,000 transcripts represented on the array, 334 genes were found to be differentially expressed in *Yap^CM−/−^* kidneys (fold change>1.27, p-value<0.05). We used both Genepaint (www.genepaint.org) and Gudmap (www.gudmap.org) databases to examine candidate expression in the developing kidney. This approach allowed us to concentrate on 24 candidates (Table S1). To confirm changes in *Yap^CM−/−^* mutants, we performed ISH and antibody staining in E14.5 control and *Yap^CM−/−^* kidneys. Our analysis confirmed that expression of Cited1, *Meox2*, *Traf1* and *Capn6* was lost in *Yap*-null CM cells (Figure 6D–6O). Similarly, expression of Pax2, *Uncx4.1* and *Sostdc1* were significantly reduced in *Yap* mutants (Figure 6A–6C, and Figure S9A–S9F). While *Fgf10* expression was barely detectable in wild-type CM cells, strong mesenchymal expression of *Fgf10* was observed in *Yap^CM−/−^* kidneys (Figure 6P–6R). Surprisingly, expression of both *Ret* and *Raldh3* was greatly increased respectively in UB tips and collecting ducts of *Yap* knockout kidneys, indicating that loss of *Yap* in the CM non-autonomously affects expression of these genes (Figure S9G–S9L). This work identifies a set of genes that depend on Yap expression during nephron development that function in differentiation and morphogenesis rather than proliferation and apoptosis.

### Inactivation of *Cdc42* phenocopies loss of *Yap*

**Figure 7:**
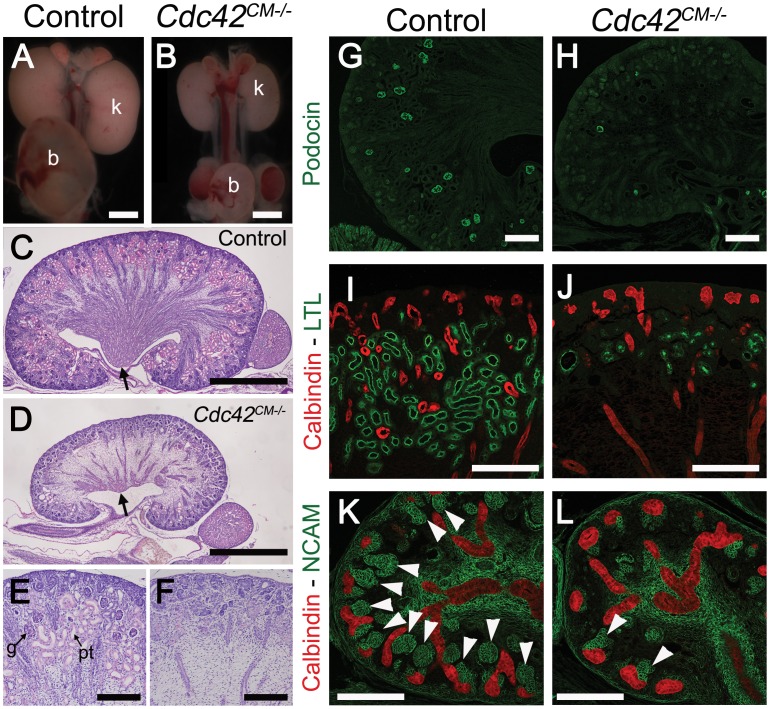
Loss of *Cdc42* phenocopies *Yap^CM−/−^* phenotype. (A,B) Macroscopic view of the urogenital system from wild-type and *Cdc42^CM−/−^*kidneys at P0. Note the reduction in kidney bladder size in mutant animals. (C–F) PAS staining (P0) from wild-type and *Cdc42^CM−/−^* animals showing smaller papilla (arrows), dramatic reduction of both CM-derived epithelial structures and glomeruli in the mutant. (G–J) Sections of P0 kidneys using late nephron-specific markers confirms the abnormal glomeruli and proximal tubules formation in *Cdc42* mutant kidneys. Glomeruli (Podocin, G,H). Proximal tubules (LTL, I,J). (K,L) NCAM staining (E15.5) reveals dramatic reduction in the number of CM-derived structures (arrowheads) in mutants compared to wild-type. k: kidney; b: bladder; g: glomeruli; pt: proximal tubule. Scale bars represent 1 mm (A–D), 200 µm (E–L).

Staining with antibodies to phospho-Yap did not indicate any obvious spatial or temporal regulation by Hippo kinases that could explain the regulation of Yap localization or activity during nephrogenesis (Figure 1B, 1E, 1F and Figure S1). We therefore searched for other potential regulators of Yap activity. Recent studies in cultured mammalian cells have demonstrated that Yap can be regulated in a Hippo kinase independent manner by mechanical signals exerted by extracellular matrix rigidity and cell shape [21]. Mechanical signals regulate Yap localization via small GTPase activity and the actin cytoskeleton. Cdc42 is a conserved and critical regulator of the actin cytoskeleton, acting through Arp2/3 and N-Wasp [33]. To examine the role of *Cdc42* in nephrogenesis, we used Six2:Cre to delete *Cdc42* from the CM population (*Cdc42^CM−/−^*). Loss of *Cdc42* from the CM resulted in a severe defect in kidney development that was strikingly similar to *Yap^CM−/−^*, with hypoplastic kidneys with empty bladders indicating lack of functional nephrons (compare Figure 7A, 7B to Figure 1G, 1H). The histology of E18.5 *Cdc42^CM−/−^* kidneys strikingly resembles that of *Yap^CM−/−^* with a distinctively reduced nephrogenic zone and a smaller papilla (Figure 7C, 7D). Convoluted renal epithelia and glomeruli were absent in the cortex of the mutant (Figure 7E, 7F). Staining with Podocin and quantification of glomeruli demonstrated a significant reduction in glomerular number in *Cdc42^CM−/−^* (glomeruli number per section at P0: control:51±2; *Cdc42^CM−/−^*:4±2; ***p<0.001), similar to that seen in *Yap^CM−/−^* kidneys (Figure 7G, 7H). *Cdc42^CM−/−^* kidneys also have fewer and truncated proximal tubules with barely discernable lumens (Figure 7I, 7J). Similar to *Yap^CM−/−^*, nephrogenic precursors are present in *Cdc42^CM−/−^* (seen by PAS staining, Six2, Sall1 and WT1 expression; Figure S10A–S10H), but the capacity of these cells to undergo nephrogenesis is dramatically reduced (NCAM staining - Figure 7K, 7L and Figure S10A, S10B). Together these data show a remarkable similarity between the effects of loss of *Yap* and the loss of *Cdc42* in the CM, suggesting they might function together in kidney development.

### Cdc42 is necessary for Yap localization

**Figure 8:**
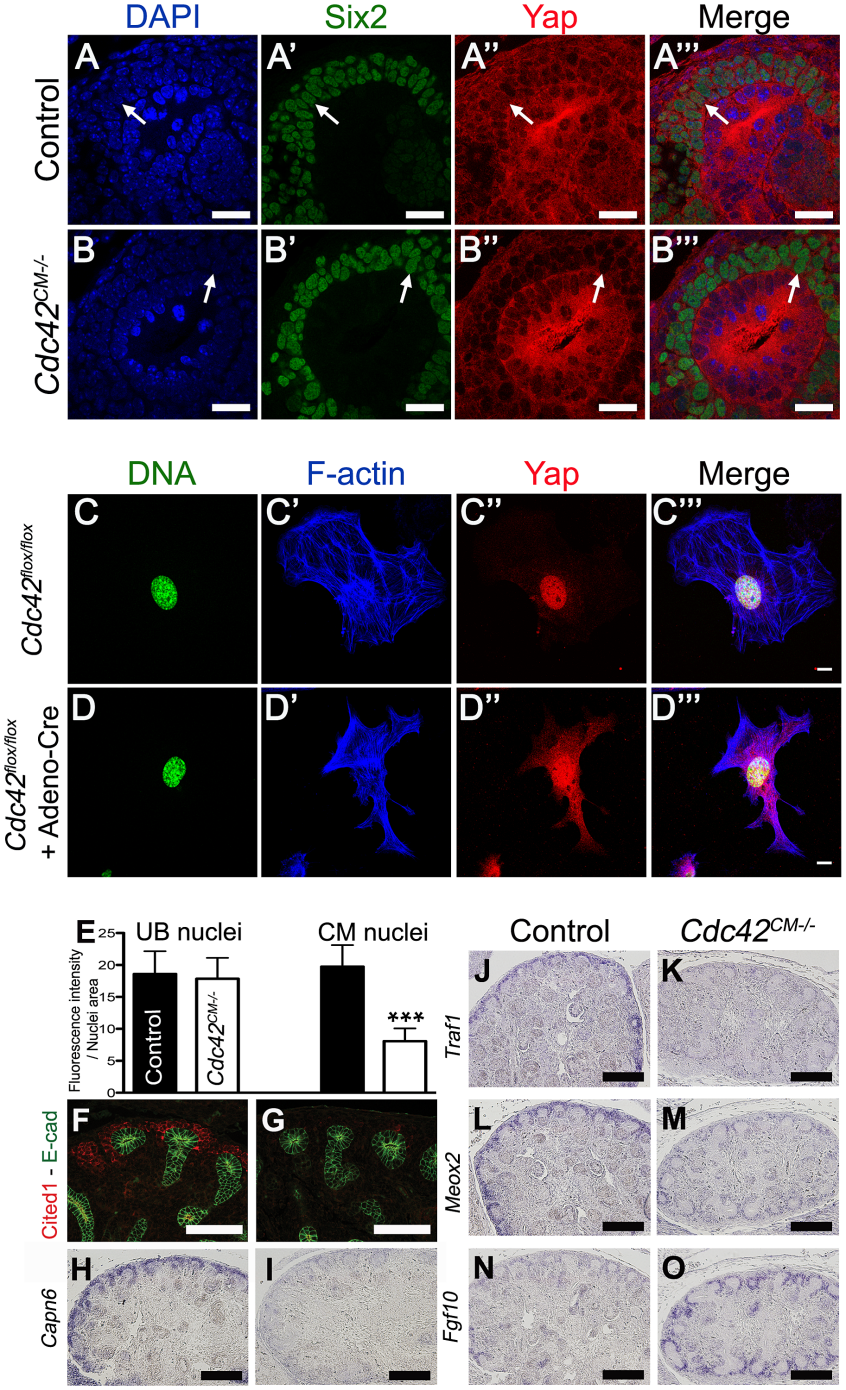
*Cdc42* is necessary for Yap to be normally localized and active. (A–B′″) Staining for Six2 and Yap shows reduce nuclear Yap staining in most of the Six2 positives cells (arrows) of *Cdc42^CM−/−^* compared to wild-type at E12.5. Control (C–C′″) and Cre infected (D–D′″) *Cdc42^flox/flox^* mouse embryonic fibroblasts (MEFs) stained with Yap antibody and doubly counterstained with phalloidin and Hoechst 33258. (E) Quantification from panels A–B′″ of Yap nuclear staining in CM and UB cells from controls (black columns) and *Cdc42^CM−/−^* (white columns) kidneys at E12.5. Data represent mean fluorescence intensity per nucleus area (100 nuclei for each genotype - ***p<0.0001). (F–M) Expression of Cited1 (F), *Capn6* (H), *Traf1* (J), *Meox2* (L) in control E14.5 kidneys, demonstrating expression in nephron progenitor cells. *Cdc42* deletion results in loss of expression of these genes in CM cells (G, I, K, M), similar to what is seen in *Yap^CM−/−^* mutant. (N,O) *IS*H reveals increase in levels of *Fgf10* expression specifically in CM cells of mutant kidneys compared to wild-type controls. Scale bars represent 25 µm (A–B′″), 10 µm (C–D′″), 100 µm (F,G), 200 µm (H–O).

The primary mechanism of regulating Yap activity is controlling Yap nuclear localization. We therefore tested if loss of *Cdc42* affected Yap nuclear localization in developing kidneys. Detailed examination of *Cdc42^CM−/−^* kidneys revealed reduced nuclear Yap in Six2 positive CM cells at E12.5 (Figure 8A–8B′″). Quantification using ImageJ software further confirmed a significant decrease of nuclear Yap in mutant CM cells compared to wild-type, while no change in the levels of Yap were observed in the nuclei of adjacent UB cells (Figure 8E).

The small GTPase RhoA has been shown to regulate Yap nuclear localization in mammalian tissue culture [21], [22], however no studies to date have examined the effects of Cdc42 on Yap localization. To better visualize changes in Yap localization upon removal of *Cdc42*, we examined cultured mouse embryonic fibroblasts (MEFs) isolated from E13.5 *Cdc42^flox/flox^* embryos. *Cdc42^flox/flox^* MEFs were infected with an adenovirus expressing Cre. Yap staining is predominantly in the nucleus in isolated control MEFs (Figure 8C–8C′″), while removal of *Cdc42* in MEFs results in more diffuse Yap staining, with reduced nuclear accumulation (Figure 8D–8D′″, lower magnification in Figure S11). Thus, loss of *Cdc42* in MEFs, as in embryonic kidneys, leads to a decrease in nuclear Yap, indicating that Cdc42 function is necessary for Yap to be normally localized in the nucleus.

### Loss of *Cdc42* leads to loss of Yap-dependent gene expression

The remarkable phenotypic similarities of *Yap^CM−/−^* and *Cdc42^CM−/−^*, coupled with the observation that loss of *Cdc42* leads to reduced levels of nuclear Yap, suggested the hypothesis that Cdc42 is necessary for Yap-dependent gene expression. We tested this hypothesis by examining expression of Yap-dependent genes in *Cdc42^CM−/−^*, by immunofluorescence and ISH. Staining of E14.5 *Cdc42^CM−/−^* kidneys revealed dramatic loss of Cited1, *Capn6*, and *Traf1*, and a clear reduction of Pax2, *Uncx4.1* and *Meox2* (Figure 8F–8M and Figure S10I–S10L). In addition, there were marked increases in *Fgf10* expression in *Cdc42^CM−/−^* mutants (Figure 8N–8O). All these changes mimicked the changes seen in *Yap* mutants (Figure 6). Thus, defective nuclear Yap localization in *Cdc42* mutants results in a loss of Yap-dependent gene expression. These data support a model where Cdc42 function is necessary for proper Yap localization and activity to control early nephron formation.

### *Yap* and *Taz* have distinct functions during nephron development

As described above, *Yap^CM−/−^* mice have dysplastic kidneys with minimal nephrogenesis. The *Yap* paralogue *Taz* is required for proper kidney development since *Taz^−/−^* mice have cystic kidneys [13],[14].

To investigate the function of *Taz* in the Six2 progenitor cells, we generated *Six2:Cre^TGC/+^ Taz^flox/flox^* mice (*Taz^CM−/−^*). Macroscopic analysis of *Taz^CM−/−^* urogenital systems shows functional kidneys (bladder filled with urine) similar in size to controls, with spotty hemorrhages at P0 (Figure 9B). Histology at P0 reveals highly cystic tubules in the cortex of *Taz^CM−/−^* kidneys (Figure 9F, 9J), similar to *Taz^−/−^* mutants. Importantly, neither the number of Six2-positive progenitor cells present at birth (Figure 9V) nor the mesenchymal-epithelial transition (NCAM - Figure 9R) were changed in *Taz^CM−/−^* mutants. Furthermore, the number of glomeruli (data not shown) remained unaltered in the *Taz^CM−/−^* mutants when compared to littermate controls indicating that *Yap* and *Taz* have distinct functions during the process of nephron formation.

To determine if the residual glomeruli and proximal tubules that form in *Yap^CM−/−^*kidneys are due to a low level redundancy by *Taz*, we generated *Six2:Cre^TGC/+^ Taz^flox/flox^ Yap^flox/flox^* mice. Significantly, *Taz^CM−/−^;Yap^CM−/−^* double mutants showed no exacerbation of glomeruli or proximal tubules deficits relative to *Yap* mutant kidneys (Figure 9H–9P and data not shown). However, some of the few proximal tubules that formed were cystic (Figure 9L, 9P), similar to *Taz* single mutants. Together, these data indicate that *Yap* and *Taz* play distinct roles during nephrogenesis.

## Discussion

*Yap* and *Taz* have well-described roles in the regulation of cell proliferation and apoptosis. Here we show that loss of *Yap* leads to defects in nephron formation and morphogenesis during renal development. We demonstrate that these defects occur independent of major changes in apoptosis or proliferation, and identify a novel set of Yap-dependent genes implicated in morphogenesis. We further show that Cdc42 function is necessary for Yap to be correctly localized in developing CM cells and in cultured MEFs, and that loss of *Cdc42* disrupts nephron formation and abolishes Yap-dependent gene expression. We propose a model in which Yap localization occurs in response to Cdc42-dependent signals, leading to expression of Yap-dependent genes during nephron development.

### *Yap* and *Taz* are necessary for distinct programs in nephron formation

*Yap* and *Taz* are closely related transcriptional co-activators that have been shown in many systems to have similar, and at times partially redundant roles in control of cell proliferation and apoptosis. A striking finding of our *in vivo* analysis is that loss of *Yap* leads primarily to misregulation of genes involved in cell fate and morphogenesis. Another surprising finding in our study was the discovery that *Yap* and *Taz* play distinct roles during kidney development. While loss of *Yap* leads to reduced nephrogenesis, with clear morphological defects at the SSB stage, loss of *Taz* leads to normal sized kidneys, with functioning nephrons, as indicated by a full bladder at birth. Proximal tubules in the *Taz* mutants are cystic, while the proximal tubules in *Yap* mutants have barely discernable lumens. Moreover, *Taz^CM−/−^;Yap^CM−/−^* double mutants show both loss of glomeruli and proximal tubules, with some tubules becoming dramatically dilated - underscoring the independence of the *Taz* and *Yap* phenotypes.

### Yap function in early nephron formation is independent of proliferation and apoptosis

Surprisingly, we did not detect any significant changes in proliferation or apoptosis in *Yap* mutants indicating that in nephrogenesis *Yap* is functioning independently of previously described roles. We did not detect any spatial regulation of Hippo-dependent Yap phosphorylation. Moreover, we found that both *Sav1* and *Mst1*/2 (*Pax3:Cre^tg/+^ Mst1^−/−^ Mst2^flox/flox^)* knockout kidneys were superficially normal (Figure S12). Further studies are needed to fully ascertain the contribution of Mst and Lats kinases to nephron development.

Although *Yap* is not essential for proliferation or apoptosis in early nephron development, we cannot exclude a later role for *Yap* in cell proliferation in the tubules. In fact the extremely short tubule segments seen in *Yap* mutants may reflect a role for *Yap* in later proliferation. Consistent with this possibility, we found that forced overexpression of Yap in adult kidneys leads to increased proliferation (data not shown). This later role of *Yap* during nephrogenesis may be controlled by the Hippo pathway, but further examination is required to examine this possibility.

### Yap-dependent genes are involved in cell signaling and morphogenesis

Using microarray analysis, we identified a number of novel Yap-dependent genes that function in nephrogenesis. Unexpectedly, these genes were not involved in control of apoptosis and proliferation, but instead involved in cell fate and morphogenesis. A subset of these genes are involved in controlling cell shape and the cytoskeleton. *Capn6*, for example, bundles and stabilizes microtubules [34]. Other genes, such as *Sostdc1* and *Fgf10* are involved in cell-cell signaling, whereas others such as *Meox2* and *Cited1* are markers of the early steps of nephrogenesis, and linked to stem cell renewal. *Yap* has been previously linked in multiple systems with stem cell proliferation and stem cell pluripotency ([16], [35], reviewed in [23]). Since Six2 expression is largely unaltered in *Yap* mutants, the observed defects lie downstream of commitment to a progenitor fate, but upstream of nephron formation.

Interestingly, of the genes we identified as Yap-dependent in nephrogenesis, only *Pax2* is absolutely required for kidney development [36]. Thus, there is likely redundancy in the morphogenesis mediated by Yap-dependent gene expression, and more complex approaches, such as double or triple knockout may be needed to uncover the critical gene programs needed for each step of nephron formation.

While removal of *Yap* leads to a dramatic loss of Cited1, *Meox2*, *Capn6* and *Traf1*, and reduction of Pax2 and NCAM, removal of *Yap* has little effect on Six2, Sall1, WT1 or *Gdnf* expression in the CM. This indicates that *Yap* deletion does not result in a loss of the CM cell population. Instead *Yap* is needed for proper CM differentiation. Interestingly, expression of the stromal markers *Raldh2* and Foxd1 was unaltered in *Yap* mutants, suggesting that observed changes in the CM of *Yap* mutants are not due to a loss of the CM cells to the stromal lineage (Figure S13). Moreover, loss of *Yap* does not primarily affect survival or proliferation of nephrogenic precursors, as no change in apoptosis and BrdU incorporation could be seen in Six2-positive cells. Thus this analysis indicates that genes required for progenitors cell survival and self-renewal are separable from those involved in their differentiation.

Some MET occurs even in the absence of *Yap*, but functional nephrons failed to form, with defects clearly visible at the SSB stages. We found that there is a dynamic pattern of nuclear Yap during early nephrogenesis. Yap localizes mainly to the nucleus in the proximal RV and the most distal and proximal cells of the SSB. Additional analysis using early nephron-specific Cre lines will be required to distinguish if changes in gene expression of progenitors cells are responsible for defective nephron formation or if Yap is needed for proper gene expression in RV and SSB.

In parallel to nephron formation, signals from the CM also promote branching morphogenesis. Our analysis of branching morphogenesis revealed that branching in *Yap* mutants slowed at E16.5 resulting in a 30% decrease in UB tips at birth. The increased *Fgf10* and *Ret* may partially compensate for the loss of CM genes seen in *Yap* mutants to maintain branching morphogenesis.

### A speculative model for Yap regulation by the Cdc42 and the cytoskeleton during kidney development

We found that *Cdc42* function is necessary for Yap to be localized normally both *in vivo* and *in vitro*, and that loss of *Cdc42* phenocopies loss of *Yap*, and abolishes Yap-dependent gene expression. How could *Cdc42* affect Yap localization and activity? *Cdc42* has conserved roles in the regulation of cell polarity and in the organization of the actin cytoskeleton. We found that neither loss of *Yap* nor *Cdc42* alters cell polarity in the developing nephron, suggesting that *Cdc42*-dependent cell polarity is not the primary mechanisms to control Yap localization. Instead, we favor a model in which the loss of *Cdc42* affects Yap localization via alterations in the cytoskeleton. Yap relocalization by mechanical stresses has been shown to be dependent on an intact actin cytoskeleton. *Cdc42* regulates the actin cytoskeleton, in part via Neuronal-Wiskcott Aldrich Syndrome protein (N-WASP) [37]. Interestingly, we found that *N-Wasp^CM−/−^* kidneys (Figure S14) were hypoplastic, with significant loss of glomeruli and proximal tubules reminiscent of the defects of *Yap* and *Cdc42* deficient kidneys.

Studies in cultured mammalian cells have demonstrated that Yap nuclear localization is regulated by mechanical signals exerted by extracellular matrix rigidity and cell shape [21]. Notably, recent studies have shown that Cdc42 is essential for matrix contraction in 3D tissue culture assays. Loss of *Cdc42* may result in defects in nephrogenesis due to loss of cytoskeletal tensions between the matrix and the tubules as they form, twist and bend during nephrogenesis. We propose that the dramatic changes in cell shape as cells aggregate, epithelialize and contort during formation of nephrons, generates mechanical stresses that are sensed via the cytoskeleton, leading to changes in the nuclear localization of Yap. Once in the nucleus, Yap then promotes expression of genes that are necessary for subsequent steps in nephron formation. While this idea is appealing, clearly much more work is needed to understand how loss of *Cdc42* leads to disruption of Yap localization, and changes in Yap-dependent gene expression.

We have shown here that loss of *Cdc42* leads to loss of Yap-dependent gene expression and loss of nuclear Yap localization. Taken together, our data suggest a model in which *Cdc42* function is necessary for Yap localization and activity during development to shape functioning nephrons.

## Materials and Methods

### Mouse lines

*Cdc42^flox^* [38], *Mst1^flox^* and *Mst2* ^−/−^ [39], *N-Wasp^flox^* [40], *Pax3:Cre^tg^* ^/+^ [41], *Sav1* ^−/−^ [17] and *Six2:Cre^TGC/+^* [25] mouse strains have been described. *Yap^flox^* and *Taz^flox^* alleles were generated by inserting LoxP sites for Cre-mediated excision flanking exons 2 as described in Figure S15. All mice were maintained on a mixed genetic background. Husbandry and ethical handling of mice were conducted according to guidelines approved by the Canadian Council on Animal Care. Embryos were genotyped by standard PCR protocol. Genotyping was done by PCR using genomic DNA prepared from mouse ear punches.

### Histological and immunological analyses

Embryonic samples from timed matings (day of vaginal plug = E0.5) were collected, fixed in 4% paraformadehyde overnight at 4C, serially dehydrated and then embedded in paraffin. Microtome sections of 7 µm thickness were examined histologically via periodic acid-Schiff staining.

For immunofluorescent analysis, paraffin sections were dewaxed and re-hydrated via ethanol series. Antigen retrieval was performed by boiling the sections for 20 minutes in Antigen Unmasking Solution (H-3300, Vector). Sections were incubated for 1 hour in blocking solution (3% BSA, 10% goat serum, 0.1% Tween20 in PBS) at room temperature. Blocking solution was replaced by a solution of primary antibodies diluted in 3% BSA, 3% goat serum, 0.1% Tween20 in PBS. The following primary antibodies were used in this study: Calbindin (PC253C, Calbiochem), Cited1 (RB-9219-P0, Neomarkers), Cytokeratin (F3418, Sigma), E-cadherin (Mouse, 610181, BD Transduction Laboratories), E-cadherin (Rabbit, #3195, Cell Signaling Technology), Ezrin (sc-58758, Santa Cruz Biotechnology), FoxD1 (gift from Andrew P. McMahon (Harvard University, Cambridge), Hnf1ß (sc-2280, Santa Cruz Biotechnology), Jag1 (#2620, Cell Signaling Technology), Laminin (L9393, Sigma), LTL (FL-1321, Vector Laboratories), NCAM (C9672, Sigma), Par3 (07-330, Millipore), Pax2 (PRB-276P, Covance), Phospho-Yap (#4911, Cell Signaling Technology), PKC (sc-216, Santa Cruz Biotechnology), Podocin (P0372, Sigma), Six2 (11562-1-AP, Proteintech), Sox9 (AB5535, Chemicon), Tomato-lectin (TL-1176, Vector Laboratories), WT1 (C-19, Santa Cruz Biotechnology), Yap (sc-101199, SantaCruz Biotechnology), Yap/Taz (#8418, Cell Signaling Technology). Relevant Cy3- or FITC-conjugated secondary antibodies (Jackson Laboratories) were used for primary antibody detection. Slides were mounted using Vectashield with or without DAPI (Vector Labs). Fluorescent images were taken with a Nikon C1 plus Digital Eclipse confocal microscope.

For immunohistochemistry, the same procedure was used, with the addition of one step after the re-hydration. Slides were immersed in 3% H2O2 in PBS for 20 minutes to block endogenous peroxidases. The Yap/Taz antibody was incubated for 48 hours at 4 degrees. Then undiluted secondary antibody (EnvisionPlus from Dako) was applied to the sections for 1 hour at room temperature. Samples were washed, developed with DAB, counterstained with hematoxylin and mounted in pertex.

### Glomerulus quantification

P0 kidneys were dissected in PBS, fixed in 4% paraformaldehyde, embedded in paraffin and sectioned. Immunostaining against Podocin was performed on P0 median kidney sections and glomeruli were counted. Means were calculated per kidney and genotype. An unpaired two-tailed t-test was used to determine the statistical significance among genotypes.

### Quantification of nuclear Yap

Quantification of Yap nuclear staining was performed using Image J software. Images were imported into Image J, and, by using DAPI staining to mark the cell nuclei, nuclear Yap signal was measured. The mean signal was calculated from 100 cells for each genotype. An unpaired two-tailed t test was used to determine the statistical significance.

### BrdU incorporation

BrdU solution containing 5-Bromo-2′-deoxyuridine (10 mg/ml) was injected intraperitoneally in pregnant mice (50 mg BrdU/kg of mice) 2 to 3 hours before embryonic dissection. The samples were prepared and sectioned as described above before being incubated overnight with anti-mouse BrdU antibody (Clone Bu20a, Dako).

### TUNEL

Terminal deoxynucleotidyl transferase, mediated digoxigenin-deoxyuridine nick end labeling (TUNEL) was performed using the Roche Cell Death Detection Kit on E18.5 kidney sections.

### *In situ* hybridization

Embryos were fixed in 4% paraformaldehyde in PBS overnight at 4C, and then paraffin embedded. Further processing of the embryos and ISH were carried out as described [42]. Riboprobes for *Capn6* [34], *Raldh3* [43], *Fgf10* [44], Ret (gift from Frank Costantini), *Slc12a1* and *Slc12a3* (gift from A. Brandli), *Meox2*, *Traf1* and *Uncx4*.1 (from the SLRI Open Freezer) were used [45].

### Microarray gene expression analysis

We used IlluminaMouseWG-6 v2.0 Expression BeadChips for whole-genome expression profiling. Pregnant mice were sacrificed at E13.75, embryos collected in ice-cold PBS and immediately decapitated. Kidneys were quickly removed and flash frozen in liquid nitrogen and stored at −80°C until RNA extraction. Both kidneys from one embryo were pooled to make one mutant or control sample. RNA was extracted using RNeasy Mini Kit (Qiagen) according to the manufacturer's protocol. The quality and quantity of collected RNA samples was checked (Agilent, Bioanalyzer) prior to submitting samples for microarray analysis. Expression profiling of three mutants and three controls samples and data analysis were done at the UHN Microarray Centre in Toronto.

### Transmission electron microscopy

Dissected kidneys were fixed in 0.1 M cacodylate buffer containing 4% paraformaldehyde and 2% glutaraldehyde. Subsequently, P0 kidneys were postfixed in 1% OsO4, dehydrated, and embedded in Quetol-spurr resin. Ultrathin resin sections stained with uranyl acetate and lead citrate were viewed using an FEI CM100 transmission electron microscope (FEI, Hillsboro, OR).

### Yap localization in MEFs

Mouse embryonic fibroblasts (MEFs) were derived from E13.5 embryos homozygous for the floxed *Cdc42* allele and maintained in 10% fetal bovine serum supplemented DMEM. MEFs were seeded into 8-well glass culture slides (BD Falcon, Bedford, MA) that were precoated with 200 µg/mL polethyleneimine to promote cell adhesion. To establish Cdc42 null MEFs, cells were infected with Cre-expressing adenovirus (Vector Biolabs) at MOI of 100. Control (uninfected) and Cdc42 null MEFs were serum-starved overnight and stimulated with serum-containing medium for 2 h and subsequently fixed in chilled 4% PFA in PBS for 10 min. Fixed cells were permeabilized for 2 min with 0.3% Triton X-100 in PBS and stained with an anti-Yap monoclonal antibody (Santa Cruz Biotechnology, Santa Cruz, CA) followed by an Alexa-Fluor488 anti-mouse secondary antibody conjugate. Cells were doubly counterstained with Texas-Red conjugated phalloidin and the DNA-binding dye Hoechst 33258. The efficiency of *Cdc42* excision was assessed by western blot of MEF lysates probed with a Cdc42 antibody (sc-8401, Santa Cruz Biotechnology) and an α-tubulin (DM1A, Sigma Aldrich).

## Supporting Information

Figure S1 Efficiency of the *Six2:Cre* deletion on *Yap* conditional allele. Low (A–B′) and high (C–D″) magnification of Yap antibody staining in control (A,C) and *Yap* mutants (B,D) confirms complete Yap deletion within both CM cells (arrows) and early nephron (arrowheads) in the mutant (D–D″), whereas staining in the UE and stroma compartments persists. (E,F) Specificity of Yap staining is confirmed by lack of staining in negative controls. (E) Sections were stained with mouse immunoglobulin (IgG) and anti-mouse Cy3 secondary addition. (F) No primary antibody added, with anti-mouse Cy3. (G) Immunohistochemistry using Yap antibody reveals same pattern of expression as seen with IF. (H–J) Immunohistochemistry of Yap/Taz antibody on *Yap^CM−/−^*, *Taz^CM−/−^* and double *Yap;Taz* mutants demonstrating that staining seen in CM cells and early nephrons is specific to Yap in our system as it disappears in *Yap* single mutants only (H), but is still present in *Taz* mutants (I).
(TIF)

Figure S2 Efficiency of the *Six2:Cre* deletion on *Yap* conditional allele. Low (A,B) and high (C–D′″) magnification of Yap antibody in control (A,C) and *Yap* mutant (B,D) confirms complete Yap deletion within CM cells (arrows) in *Yap* mutant (D–D″), whereas staining in the UE and stromal compartments persists. (E,F) Staining specificity is demonstrated by lack of staining in relevant controls. (E) No Yap primary antibody, but inclusion of anti-rabbit Cy3. (F) Staining with rabbit immunoglobulin and anti-rabbit Cy3.
(TIF)

Figure S3 Loss of foot processes in *Yap* mutants. Transmission electron micrographs confirm abnormal glomeruli structure with foot process (arrows) effacement in P0 *Yap* mutant compared to controls. ec, endothelial cells, p: podocyte. Scale bars represent 10 µm (A,B) and 2 µm (C,D).
(TIF)

Figure S4 Branching morphogenesis in *Yap* mutants. Staining for Calbindin at E14.5 shows similar number of epithelial tips at E14.5 (A,B), while branching is severely decreased in hypoplastic *Yap* mutant kidneys at P0 (C,D). Slides were counterstained with DAPI. (E) Calbindin staining was used to quantify branching morphogenesis in control (black columns) and *Yap* mutant (white columns) at E14.5, E16.5 (*p = 0.0369) and P0 (***p<0.001). Scale bars represent 500 µm.
(TIF)

Figure S5 Cell polarity appears normal in *Yap* and *Cdc42* mutant. Immunostaining for Par3 and E-cadherin (A–C, P0), Ezrin (D,E, P0), LTL and Ecadherin (F,G, P0) and PKC (H–K, E15.5) reveals no defects in cell polarity in early nephrons. Scale bars represent 50 µm.
(TIF)

Figure S6 Quantification of Six2 positive cells. Quantification of progenitor cells number using Six2 antibody, at E15.5 reveals a slight but insignificant reduction in *Yap* mutants compared to controls.
(TIF)

Figure S7 *Yap* deletion impacts nephrogenesis independently of both Wnt/ß-catenin and Notch signaling pathways. (A,B) ISH analysis shows normal expression pattern of *Wnt9b* in both genotypes. (C–P) Staining for known Wnt/ß-catenin targets - *Pla2g7* (C,D), *C1qdc2* (E,F), Lef1 (G,H), *Fgf8* (I,J), *Wnt4* (K,L), *Pax8* (M,N) and *Lim1* (O,P) – reveals normal expression in control and mutant kidneys. (Q–X) ISH reveals no effect of *Yap* deletion on expression of components of the Notch pathway – *Notch2* (Q,R), *Jag1* (S,T), *Hes1* (U,V), *Hes5* (W,X). All staining performed at E14.5. Scale bars represent 100 µm.
(TIF)

Figure S8 Haploinsufficiency for ß-catenin does not alter the *Yap^CM−/−^* phenotype. (A–C) PAS staining of P0 control, *Yap^CM−/−^* and *Six2:Cre^TGC/+^ Yap^flox/flox^ ß-catenin(KO)^flox/+^*. (D–F) LTL/Calbindin staining of P0 control, *Yap^CM−/−^* and *Six2:Cre^TGC/+^ Yap^flox/flox^ ß-catenin(KO)^flox/+^*. Scale bars represent 1 mm (A–C), 200 µm (D–F).
(TIF)

Figure S9 Changes in genes expression in *Yap^CM−/−^* kidneys. (A–E) *In situ* hybridization reveals decreases in *Uncx4.1* and *Sostdc1* in *Yap* mutants compared to wild-type (E14.5). (G,K) *In situ* hybridization shows increased levels of expression of *Ret* (G,H) and *Raldh3* (J,K) in UB tips and trunk respectively in E14.5 *Yap^CM−/−^* kidneys. (C,F,I,L) Graphical representation of the microarray data of control (black colums) and *Yap* mutant (white columns). (*:p<0.05; **:p<0.001). Scale bars represent 200 µm.
(TIF)

Figure S10 Loss of *Cdc42* phenocopies loss of *Yap*. (A,B) PAS staining of E14.5 wild-type and *Cdc42^CM−/−^* kidneys showing the presence of condensing mesenchymal cells (arrows) but dramatic loss of CM-derived epithelial structures (arrowheads). Immunostaining analysis for Six2 (C,D), Sall1 (E,F) and WT1 (G,H) at E14.5 shows presence of CM cells in both genotypes (arrows). E-cadherin and Calbindin were used to visualize the UB compartment. (I,J) Immunostaining at E14.5 shows normal expression of Pax2 in the UB, but decreased expression in the *Cdc42*-deficient CM cells (arrows). (K,L) *In situ* hybridization at E14.5 reveals decrease in *Uncx4.1* in *Cdc42^CM−/−^* mutant. Scale bars represent 100 µm.
(TIF)

Figure S11 Lower magnification of Yap localization in MEFs and validation of Cdc42 knock down. Control (A–A′″) and Cre infected (B–B′″) *Cdc42^flox/flox^* MEFs stained with Yap antibody and doubly counterstained with phalloidin and Hoechst 33258. (C) Western-blot analysis using Cdc42 antibody reveals loss of Cdc42 protein in the Cre-infected *Cdc42^flox/flox^* MEFs versus control MEFs. Loading control assessed by using α-Tubulin antibody.
(TIF)

Figure S12 *Sav1* and *Mst1/2* removal have minor effects on kidney development. PAS staining (A,B) and NCAM-Pax2 staining (C,D) of P0 control (*Pax3:Cre^tg/+^ Mst1^flox/+^ Mst2^−/−^*) and *Pax3:Cre^tg/+^ Mst1^flox/flox^ Mst2^−/−^* showing normal histology. (E–F) Phospho-Yap staining of P0 control and *Pax3:Cre^tg/+^ Mst1^flox/flox^ Mst2^−/−^* showing comparable phospho-Yap staining in both genotypes. (G,H) PAS staining of E18.5 kidneys from wild-type and *Sav1^−/−^* animals. (I,J) E18.5 kidneys stained for proximal tubule markers (LTL) and Pax2. Scale bars represent 100 µm.
(TIF)

Figure S13 No change in stromal markers gene expression in *Yap* mutant. (A,B) ISH analysis reveals similar expression pattern of *Raldh2* in both genotype. (C,D) Antibody staining for Foxd1 shows similar expression in both genotypes. E-cadherin was used to visualize the UB compartment. Scale bars represent 100 µm.
(TIF)

Figure S14 Loss of *N-Wasp* leads to hypoplasia and loss of glomeruli and proximal tubules. (A–C) PAS staining (P0) of wild-type and *N-Wasp^CM−/−^* kidneys showing hypoplasia in mutants. (D,E) Sections of P0 kidneys processed for Podocin analysis shows a strong decrease in glomeruli formation in *N-Wasp^CM−/−^* mutant kidneys. Average count and standard deviation from four controls and four mutants are shown in F. ***p<0.0001. (G–I) Sections of P0 kidneys processed for LTL and Calbindin staining shows reduced proximal tubule formation in *N-Wasp^CM−/−^* mutant kidneys. Scale bars represent 100 µm (A–E), 200 µm (G–I).
(TIF)

Figure S15 Generation of *Yap* and *Taz* flox allele. (A) *Yap* flox allele was generated by inserting LoxP sites for Cre-mediated excision flanking exons 2. (B) The *Taz* flox allele was generated by inserting LoxP sites for Cre-mediated excision flanking exons 2. (C) Western-blot analysis using Taz antibodies reveals absence of Taz protein in the *Taz^−/−^* kidneys.
(TIF)

Table S1 Candidates genes of microarrays on E13.5 *Yap^CM−/−^* mutant kidneys compared to *Yap^flox/+^* controls. All 24 genes were assayed by ISH and/or antibody staining on E14.5 wild-type and *Yap^CM−/−^* kidneys.
(PDF)
