# Supplementary figures and images for: Yap- and Cdc42-Dependent Nephrogenesis and Morphogenesis during Mouse Kidney Development
Source: PLoS Genet. 2013 Mar 21;9(3):e1003380. doi: 10.1371/journal.pgen.1003380 (PMC3605093; doi:10.1371/journal.pgen.1003380)

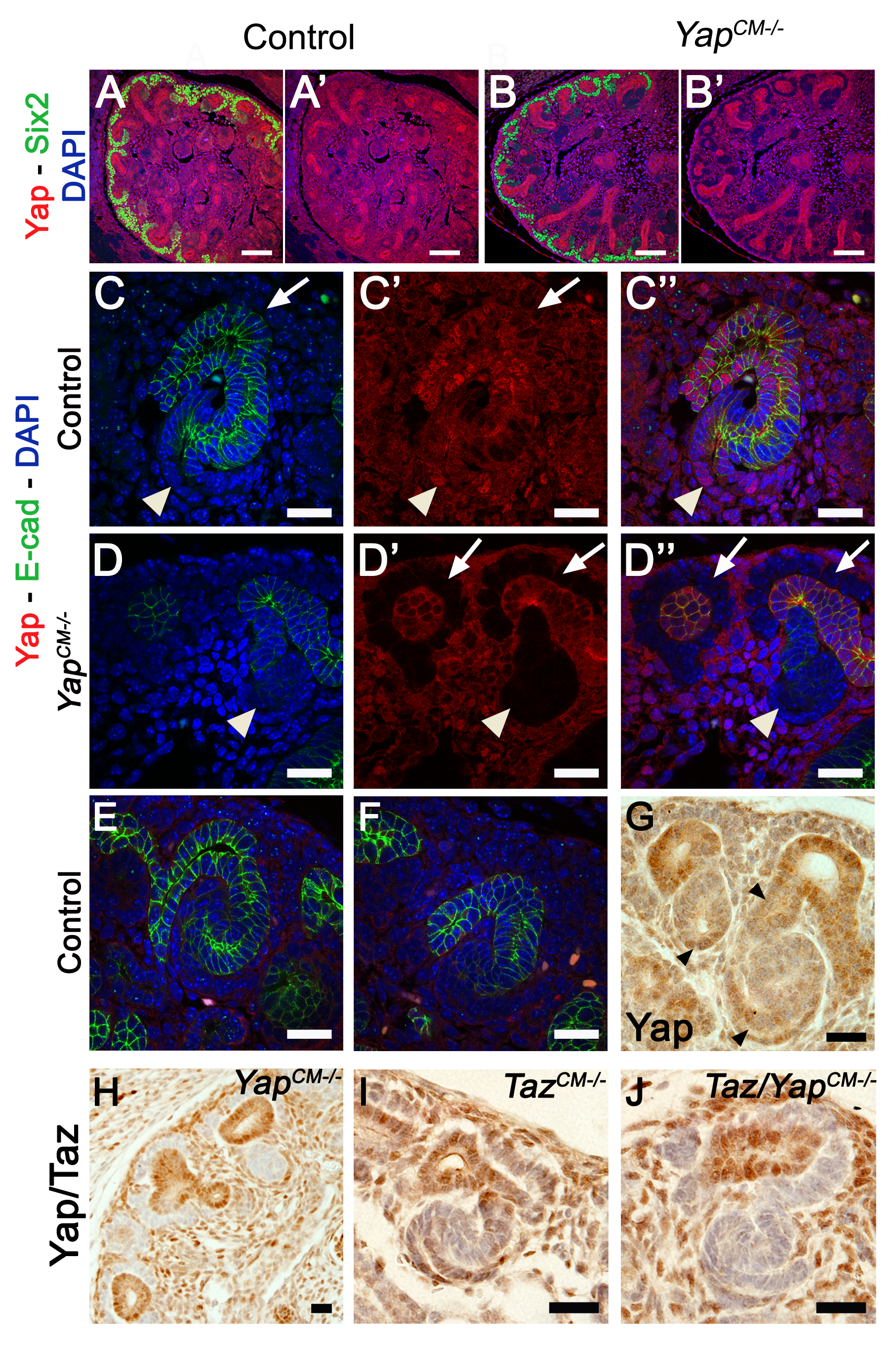

Supplement: Figure S1 — Efficiency of the Six2:Cre deletion on Yap conditional allele. Low (A–B′) and high (C–D″) magnification of Yap antibody staining in control (A,C) and Yap mutants (B,D) confirms complete Yap deletion within both CM cells (arrows) and early nephron (arrowheads) in the mutant (D–D″), whereas staining in the UE and stroma compartments persists. (E,F) Specificity of Yap staining is confirmed by lack of staining in negative controls. (E) Sections were stained with mouse immunoglobulin (IgG) and anti-mouse Cy3 secondary addition. (F) No primary antibody added, with anti-mouse Cy3. (G) Immunohistochemistry using Yap antibody reveals same pattern of expression as seen with IF. (H–J) Immunohistochemistry of Yap/Taz antibody on YapCM−/−, TazCM−/− and double Yap;Taz mutants demonstrating that staining seen in CM cells and early nephrons is specific to Yap in our system as it disappears in Yap single mutants only (H), but is still present in Taz mutants (I). (TIF) [file pgen.1003380.s001.tif]

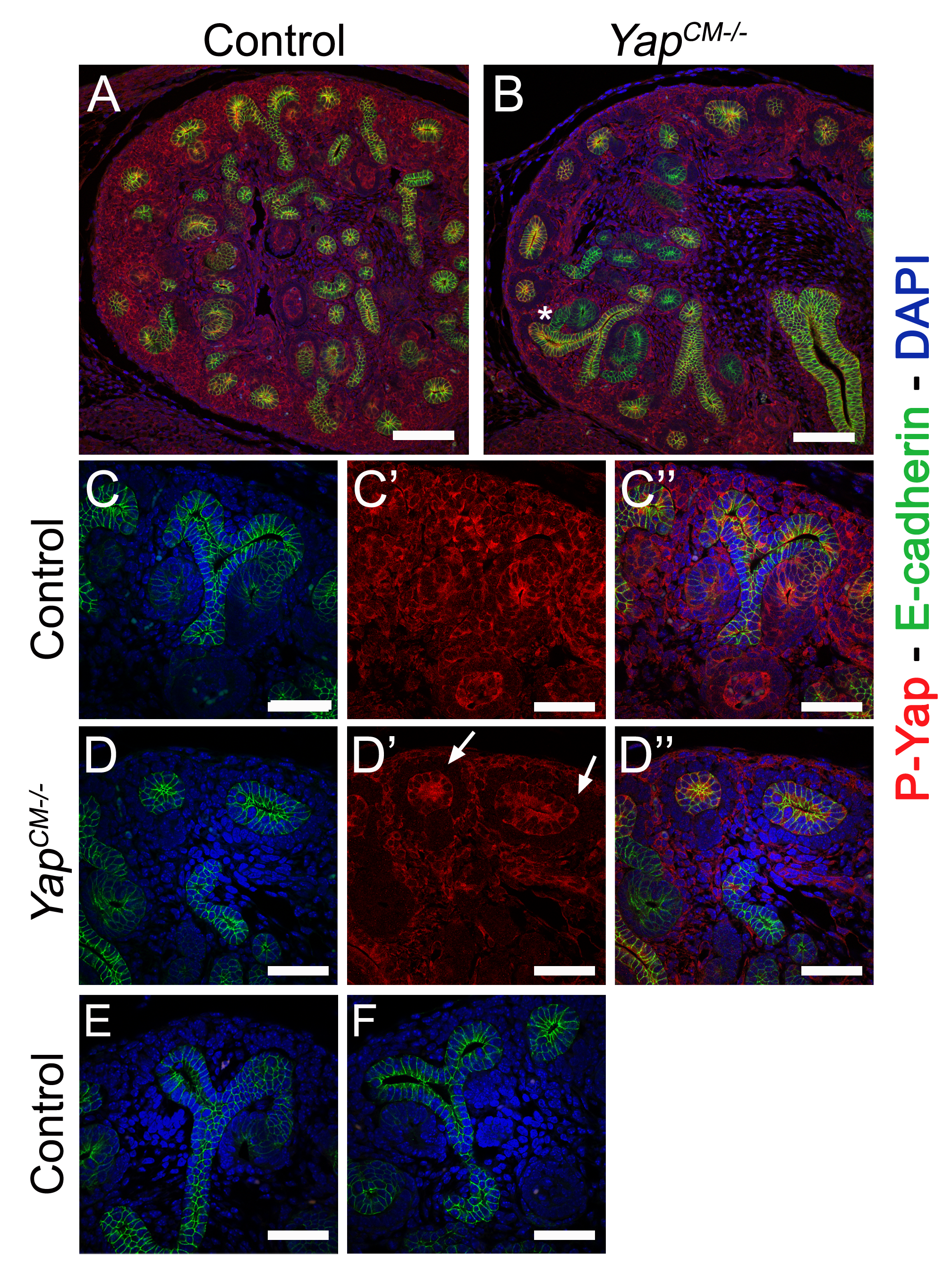

Supplement: Figure S2 — Efficiency of the Six2:Cre deletion on Yap conditional allele. Low (A,B) and high (C–D′″) magnification of Yap antibody in control (A,C) and Yap mutant (B,D) confirms complete Yap deletion within CM cells (arrows) in Yap mutant (D–D″), whereas staining in the UE and stromal compartments persists. (E,F) Staining specificity is demonstrated by lack of staining in relevant controls. (E) No Yap primary antibody, but inclusion of anti-rabbit Cy3. (F) Staining with rabbit immunoglobulin and anti-rabbit Cy3. (TIF) [file pgen.1003380.s002.tif]

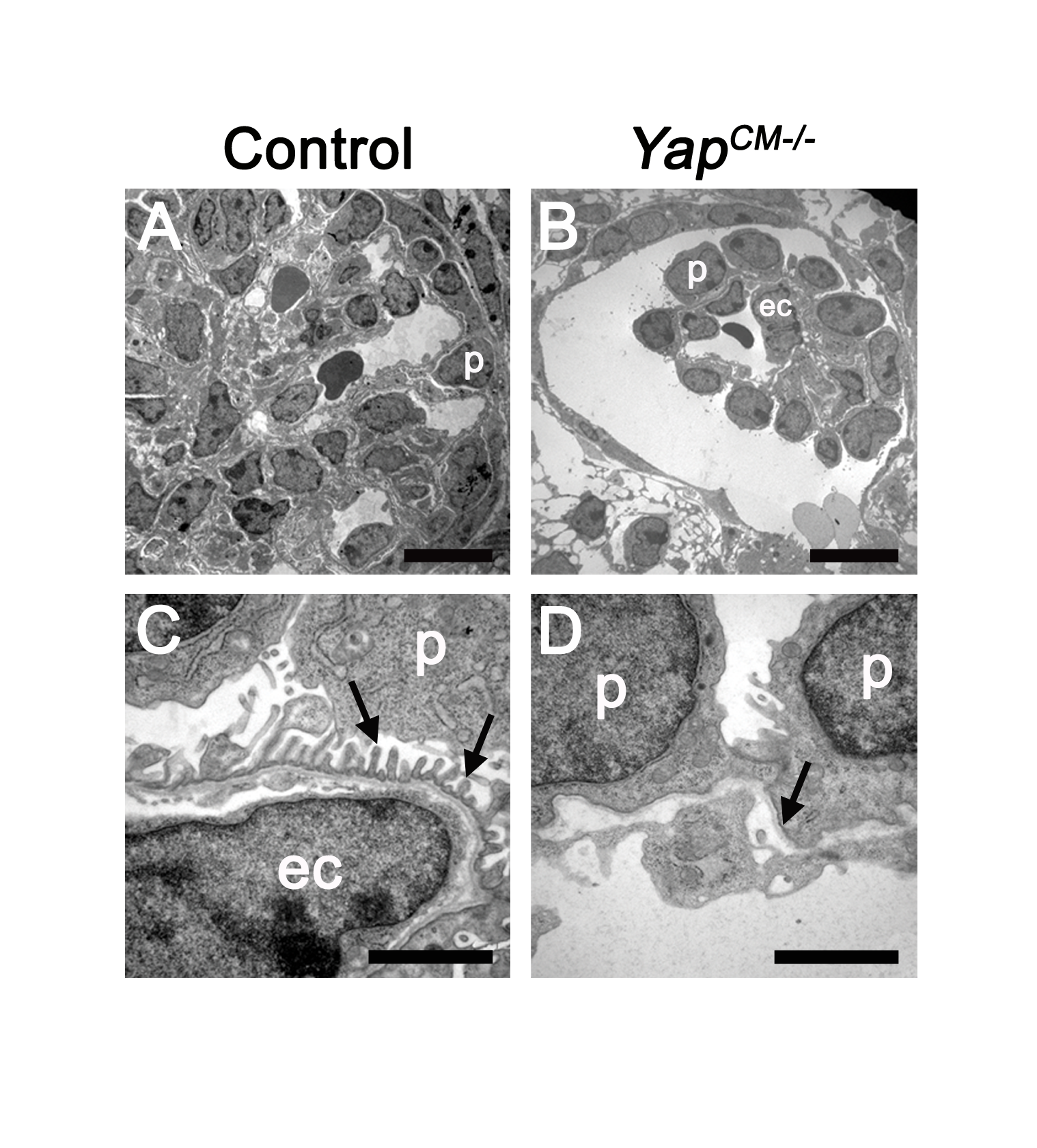

Supplement: Figure S3 — Loss of foot processes in Yap mutants. Transmission electron micrographs confirm abnormal glomeruli structure with foot process (arrows) effacement in P0 Yap mutant compared to controls. ec, endothelial cells, p: podocyte. Scale bars represent 10 µm (A,B) and 2 µm (C,D). (TIF) [file pgen.1003380.s003.tif]

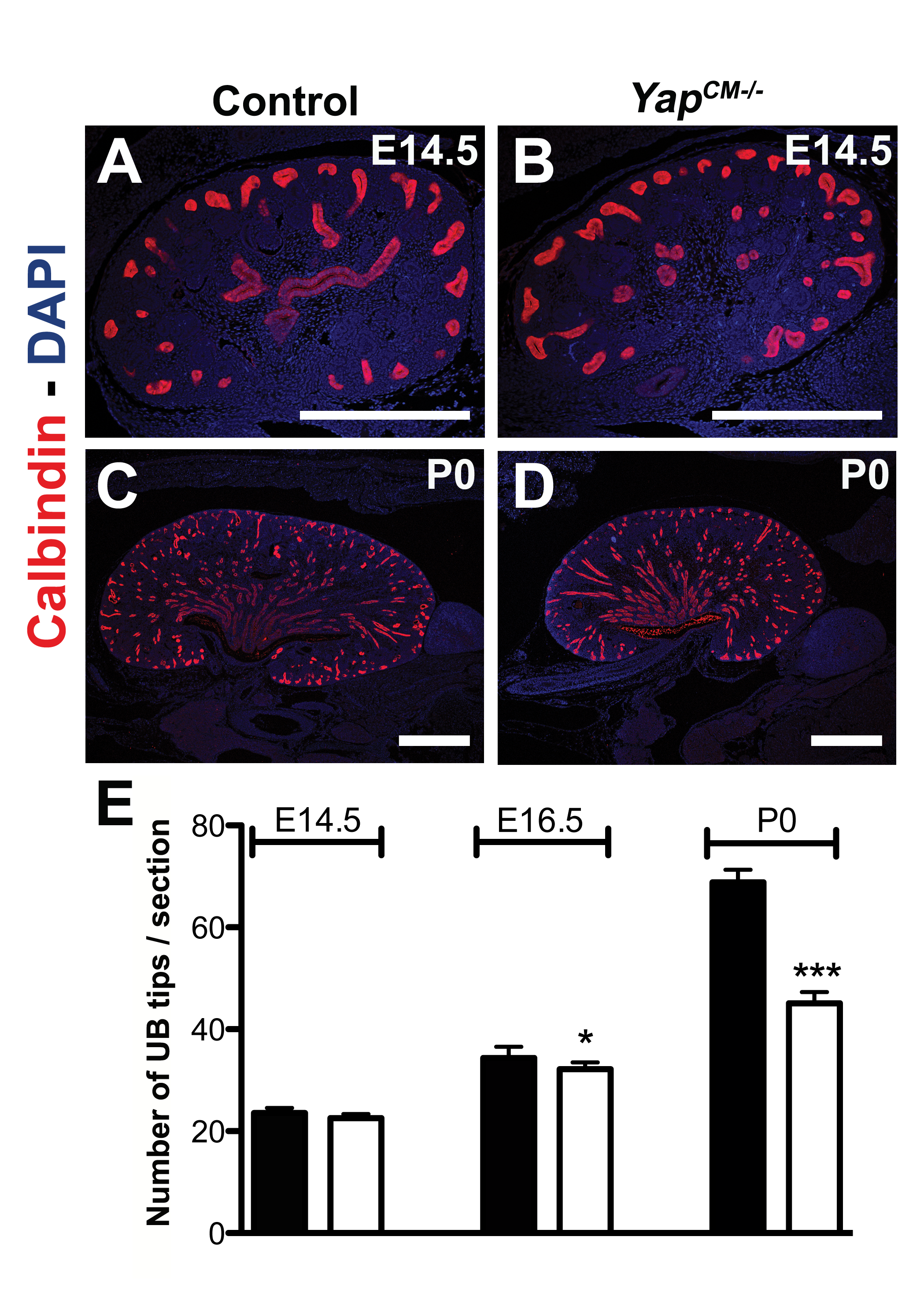

Supplement: Figure S4 — Branching morphogenesis in Yap mutants. Staining for Calbindin at E14.5 shows similar number of epithelial tips at E14.5 (A,B), while branching is severely decreased in hypoplastic Yap mutant kidneys at P0 (C,D). Slides were counterstained with DAPI. (E) Calbindin staining was used to quantify branching morphogenesis in control (black columns) and Yap mutant (white columns) at E14.5, E16.5 (*p = 0.0369) and P0 (***p<0.001). Scale bars represent 500 µm. (TIF) [file pgen.1003380.s004.tif]

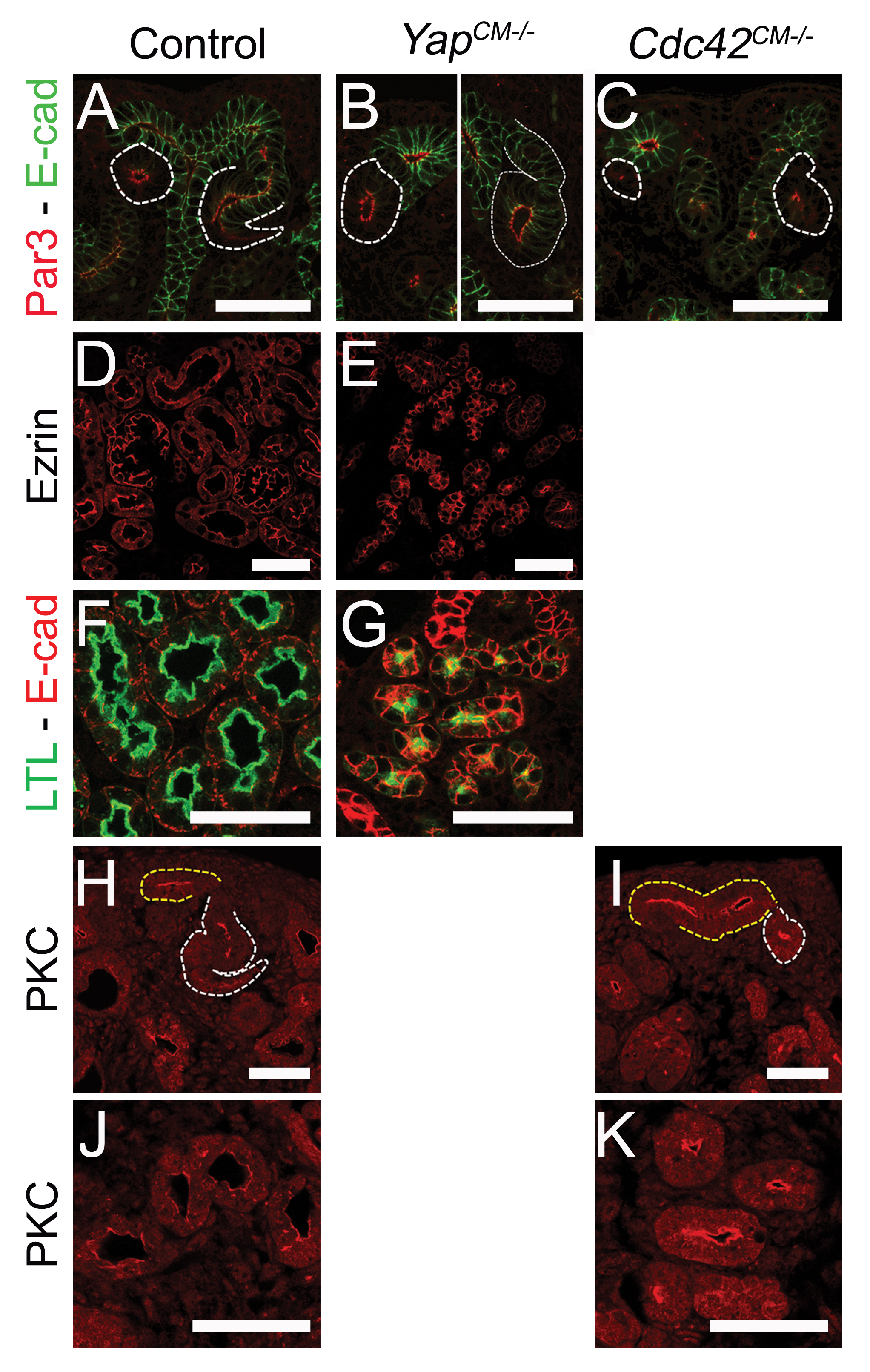

Supplement: Figure S5 — Cell polarity appears normal in Yap and Cdc42 mutant. Immunostaining for Par3 and E-cadherin (A–C, P0), Ezrin (D,E, P0), LTL and Ecadherin (F,G, P0) and PKC (H–K, E15.5) reveals no defects in cell polarity in early nephrons. Scale bars represent 50 µm. (TIF) [file pgen.1003380.s005.tif]

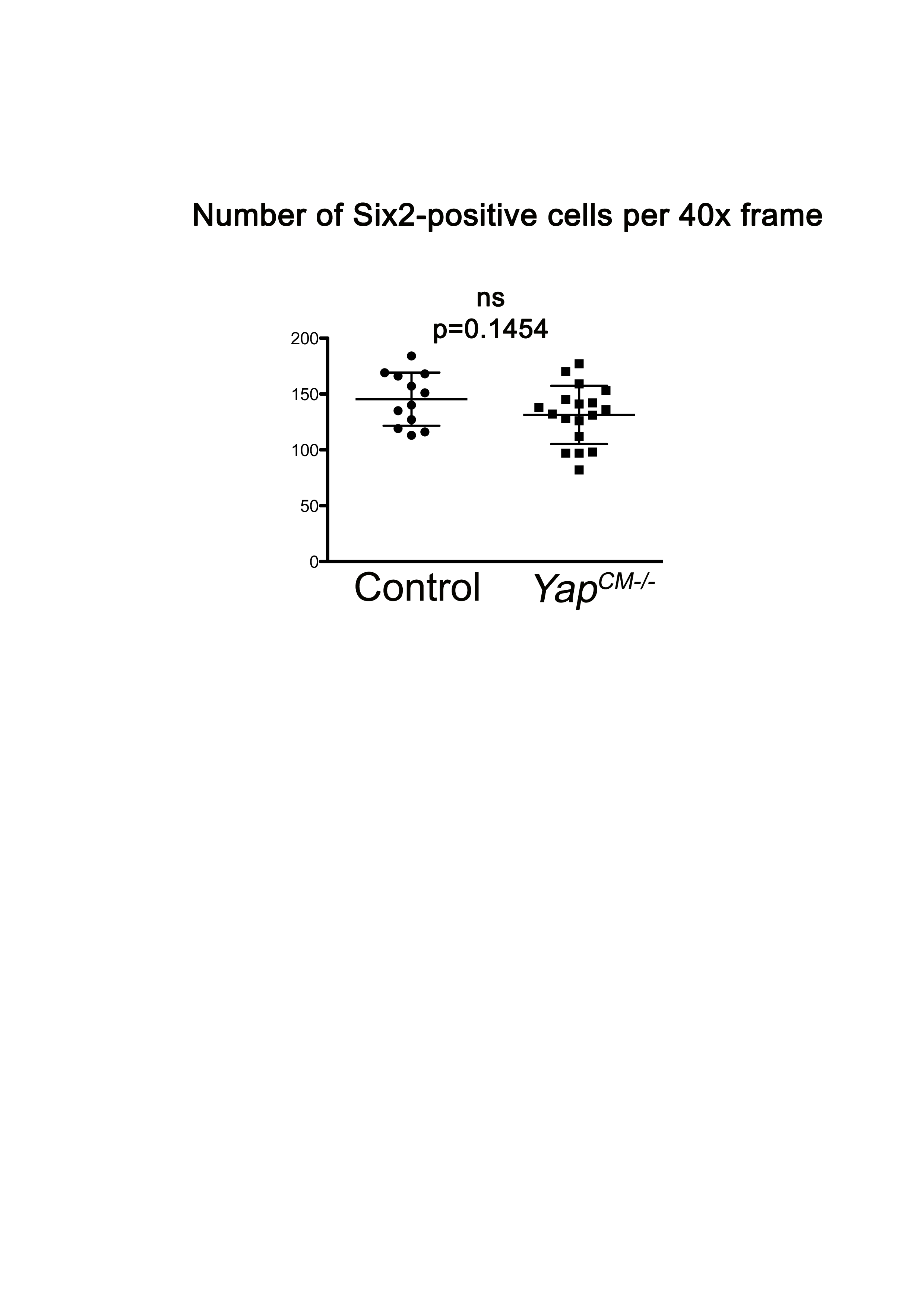

Supplement: Figure S6 — Quantification of Six2 positive cells. Quantification of progenitor cells number using Six2 antibody, at E15.5 reveals a slight but insignificant reduction in Yap mutants compared to controls. (TIF) [file pgen.1003380.s006.tif]

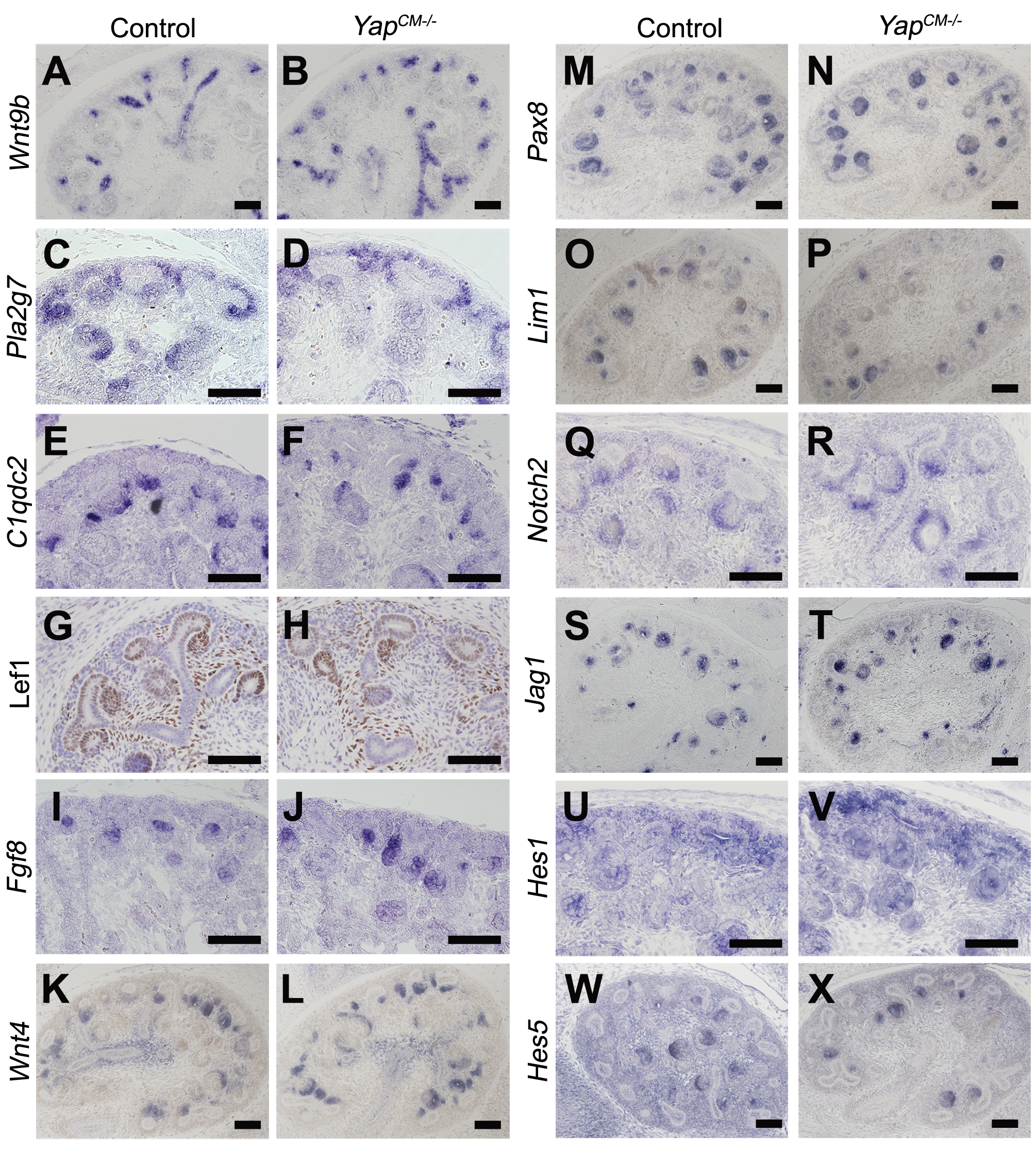

Supplement: Figure S7 — Yap deletion impacts nephrogenesis independently of both Wnt/ß-catenin and Notch signaling pathways. (A,B) ISH analysis shows normal expression pattern of Wnt9b in both genotypes. (C–P) Staining for known Wnt/ß-catenin targets - Pla2g7 (C,D), C1qdc2 (E,F), Lef1 (G,H), Fgf8 (I,J), Wnt4 (K,L), Pax8 (M,N) and Lim1 (O,P) – reveals normal expression in control and mutant kidneys. (Q–X) ISH reveals no effect of Yap deletion on expression of components of the Notch pathway – Notch2 (Q,R), Jag1 (S,T), Hes1 (U,V), Hes5 (W,X). All staining performed at E14.5. Scale bars represent 100 µm. (TIF) [file pgen.1003380.s007.tif]

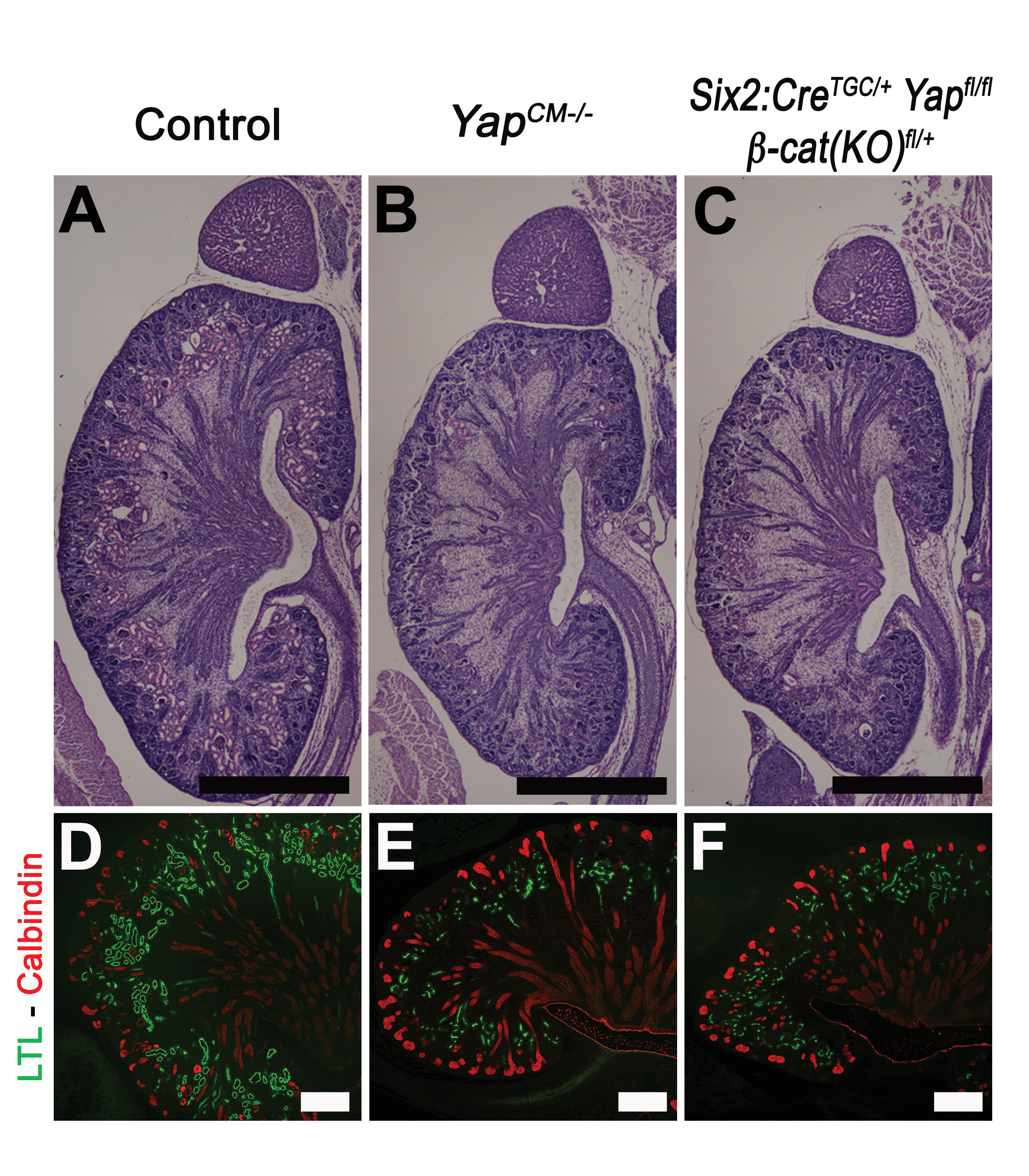

Supplement: Figure S8 — Haploinsufficiency for ß-catenin does not alter the YapCM−/− phenotype. (A–C) PAS staining of P0 control, YapCM−/− and Six2:CreTGC/+ Yapflox/flox ß-catenin(KO)flox/+. (D–F) LTL/Calbindin staining of P0 control, YapCM−/− and Six2:CreTGC/+ Yapflox/flox ß-catenin(KO)flox/+. Scale bars represent 1 mm (A–C), 200 µm (D–F). (TIF) [file pgen.1003380.s008.tif]

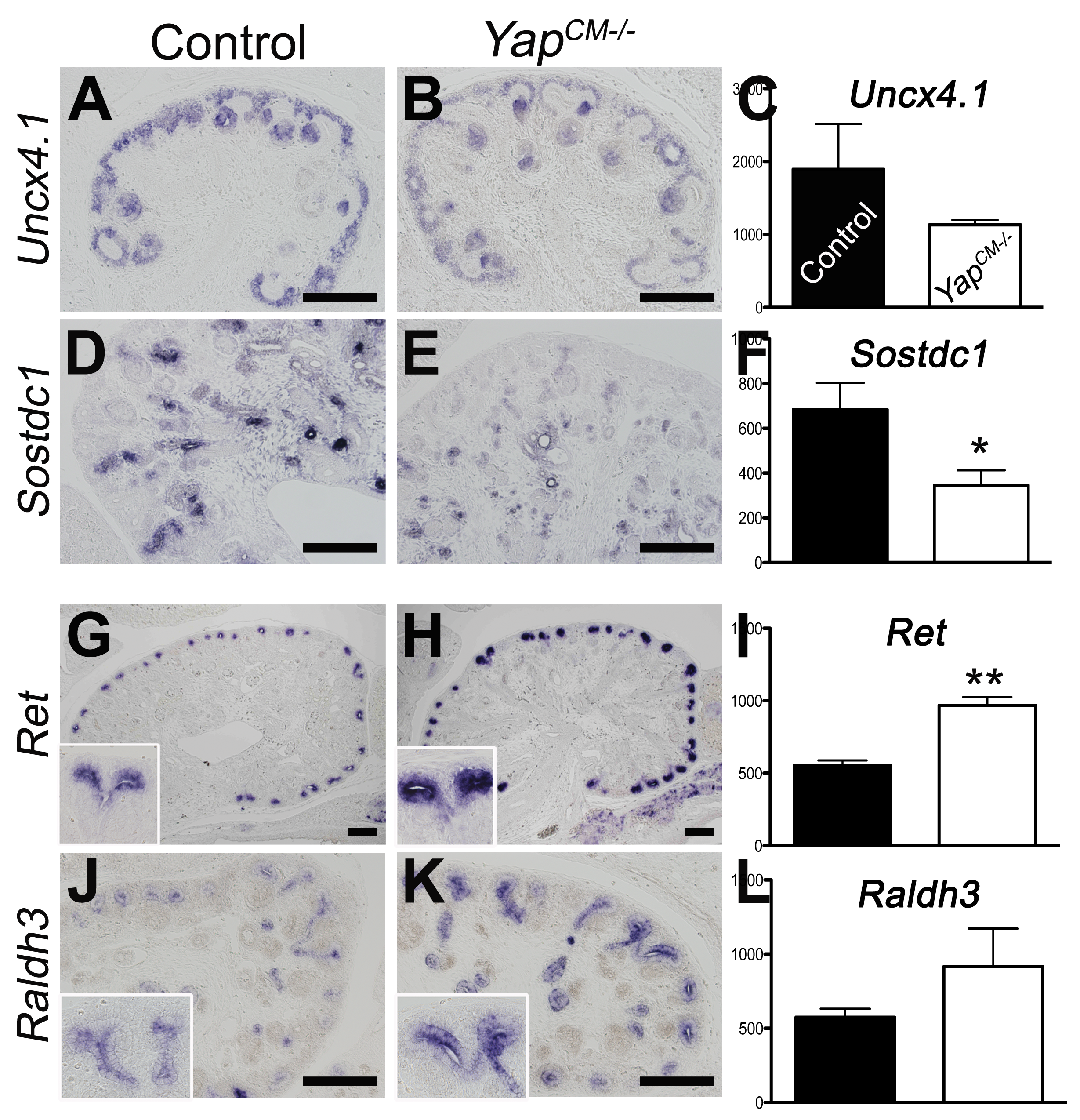

Supplement: Figure S9 — Changes in genes expression in YapCM−/− kidneys. (A–E) In situ hybridization reveals decreases in Uncx4.1 and Sostdc1 in Yap mutants compared to wild-type (E14.5). (G,K) In situ hybridization shows increased levels of expression of Ret (G,H) and Raldh3 (J,K) in UB tips and trunk respectively in E14.5 YapCM−/− kidneys. (C,F,I,L) Graphical representation of the microarray data of control (black colums) and Yap mutant (white columns). (*:p<0.05; **:p<0.001). Scale bars represent 200 µm. (TIF) [file pgen.1003380.s009.tif]

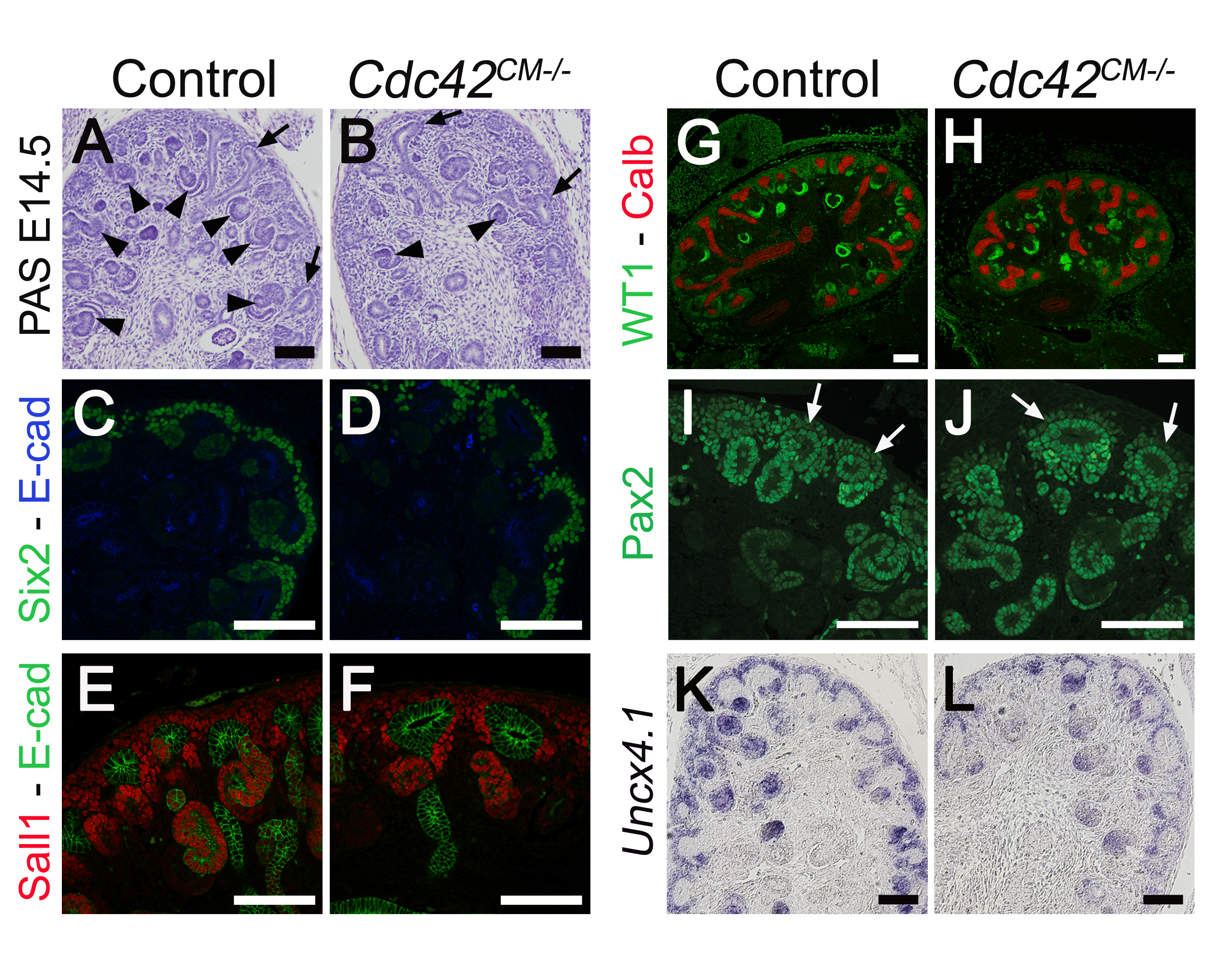

Supplement: Figure S10 — Loss of Cdc42 phenocopies loss of Yap. (A,B) PAS staining of E14.5 wild-type and Cdc42CM−/− kidneys showing the presence of condensing mesenchymal cells (arrows) but dramatic loss of CM-derived epithelial structures (arrowheads). Immunostaining analysis for Six2 (C,D), Sall1 (E,F) and WT1 (G,H) at E14.5 shows presence of CM cells in both genotypes (arrows). E-cadherin and Calbindin were used to visualize the UB compartment. (I,J) Immunostaining at E14.5 shows normal expression of Pax2 in the UB, but decreased expression in the Cdc42-deficient CM cells (arrows). (K,L) In situ hybridization at E14.5 reveals decrease in Uncx4.1 in Cdc42CM−/− mutant. Scale bars represent 100 µm. (TIF) [file pgen.1003380.s010.tif]

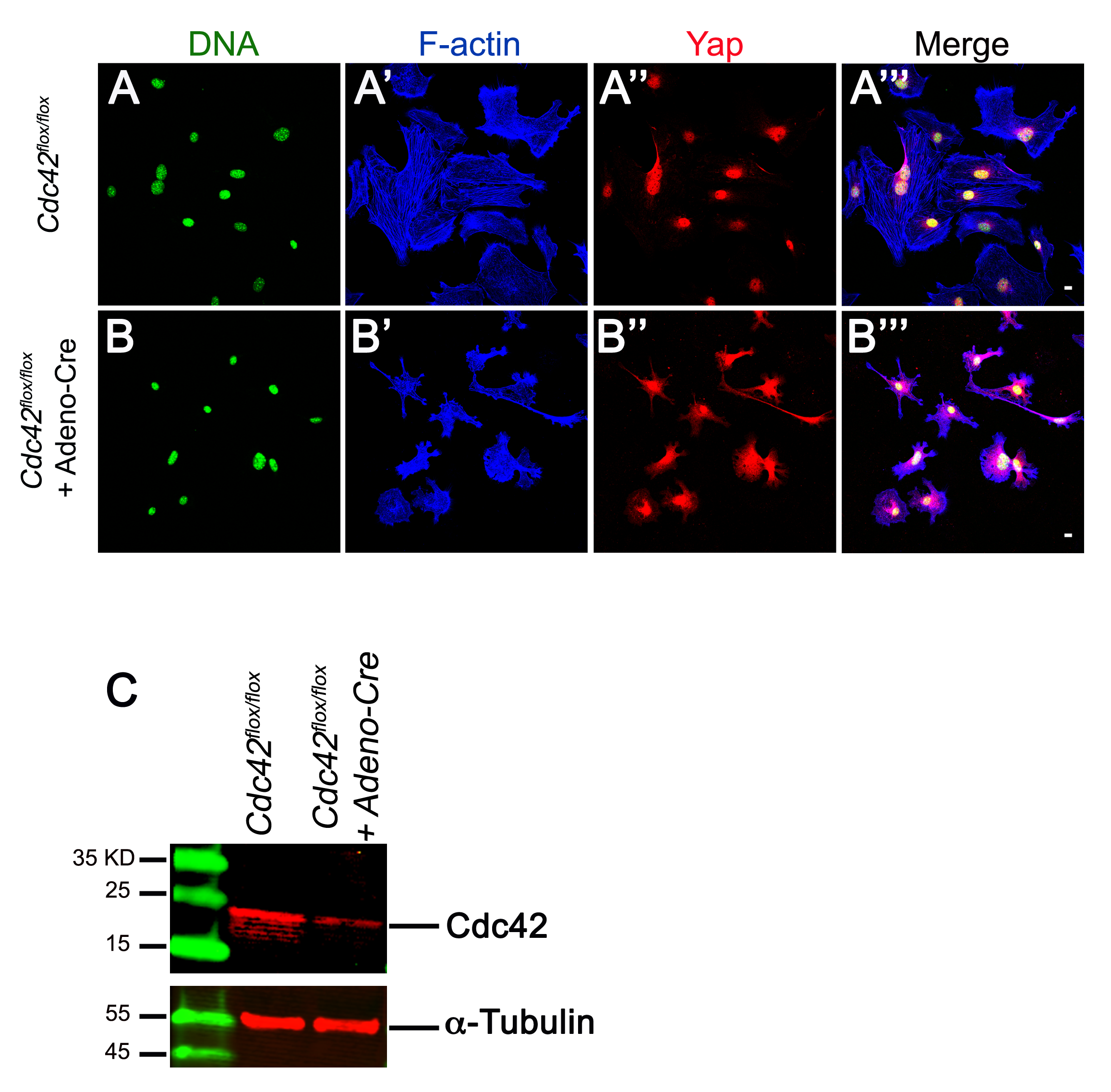

Supplement: Figure S11 — Lower magnification of Yap localization in MEFs and validation of Cdc42 knock down. Control (A–A′″) and Cre infected (B–B′″) Cdc42flox/flox MEFs stained with Yap antibody and doubly counterstained with phalloidin and Hoechst 33258. (C) Western-blot analysis using Cdc42 antibody reveals loss of Cdc42 protein in the Cre-infected Cdc42flox/flox MEFs versus control MEFs. Loading control assessed by using α-Tubulin antibody. (TIF) [file pgen.1003380.s011.tif]

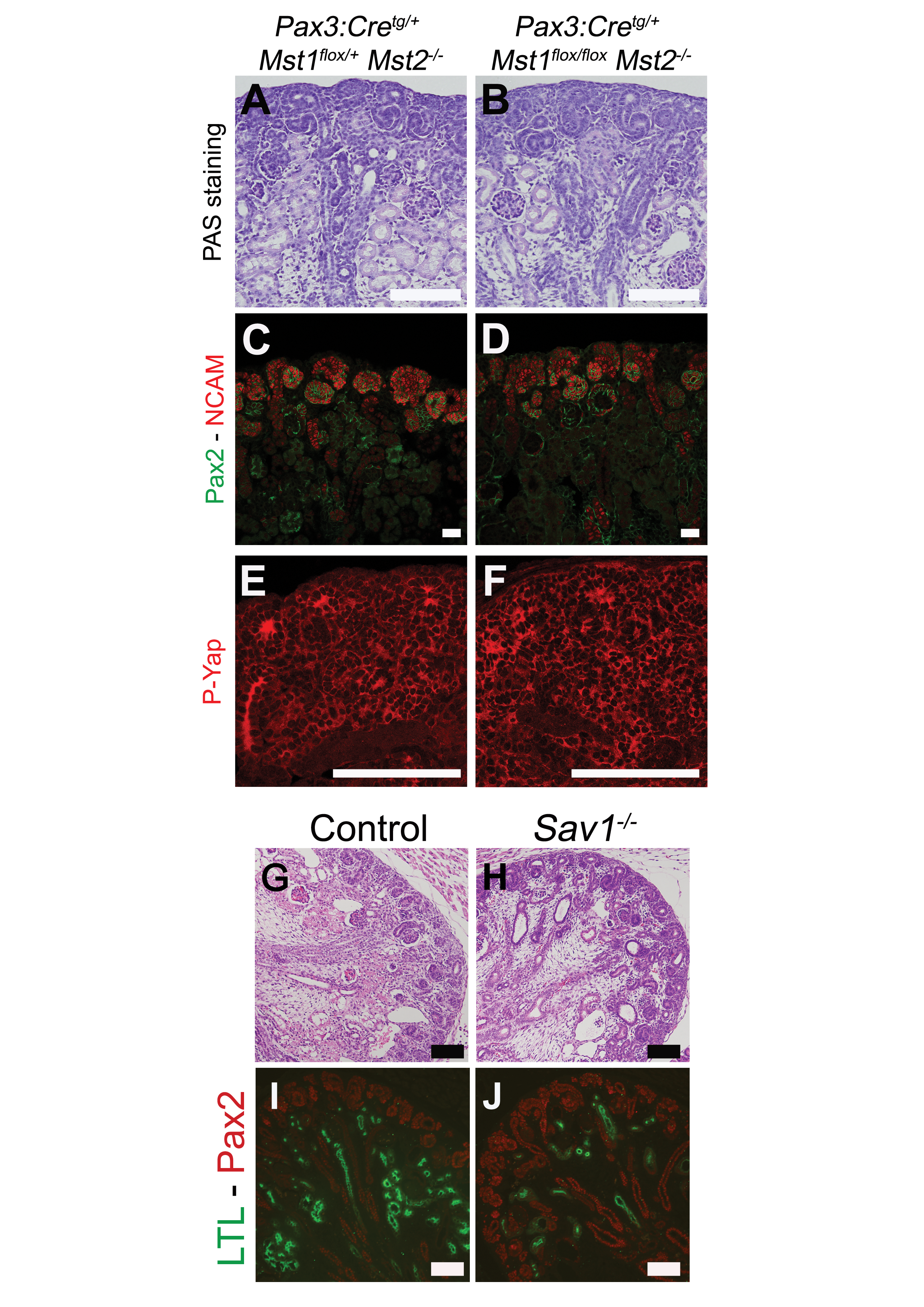

Supplement: Figure S12 — Sav1 and Mst1/2 removal have minor effects on kidney development. PAS staining (A,B) and NCAM-Pax2 staining (C,D) of P0 control (Pax3:Cretg/+ Mst1flox/+ Mst2−/−) and Pax3:Cretg/+ Mst1flox/flox Mst2−/− showing normal histology. (E–F) Phospho-Yap staining of P0 control and Pax3:Cretg/+ Mst1flox/flox Mst2−/− showing comparable phospho-Yap staining in both genotypes. (G,H) PAS staining of E18.5 kidneys from wild-type and Sav1−/− animals. (I,J) E18.5 kidneys stained for proximal tubule markers (LTL) and Pax2. Scale bars represent 100 µm. (TIF) [file pgen.1003380.s012.tif]

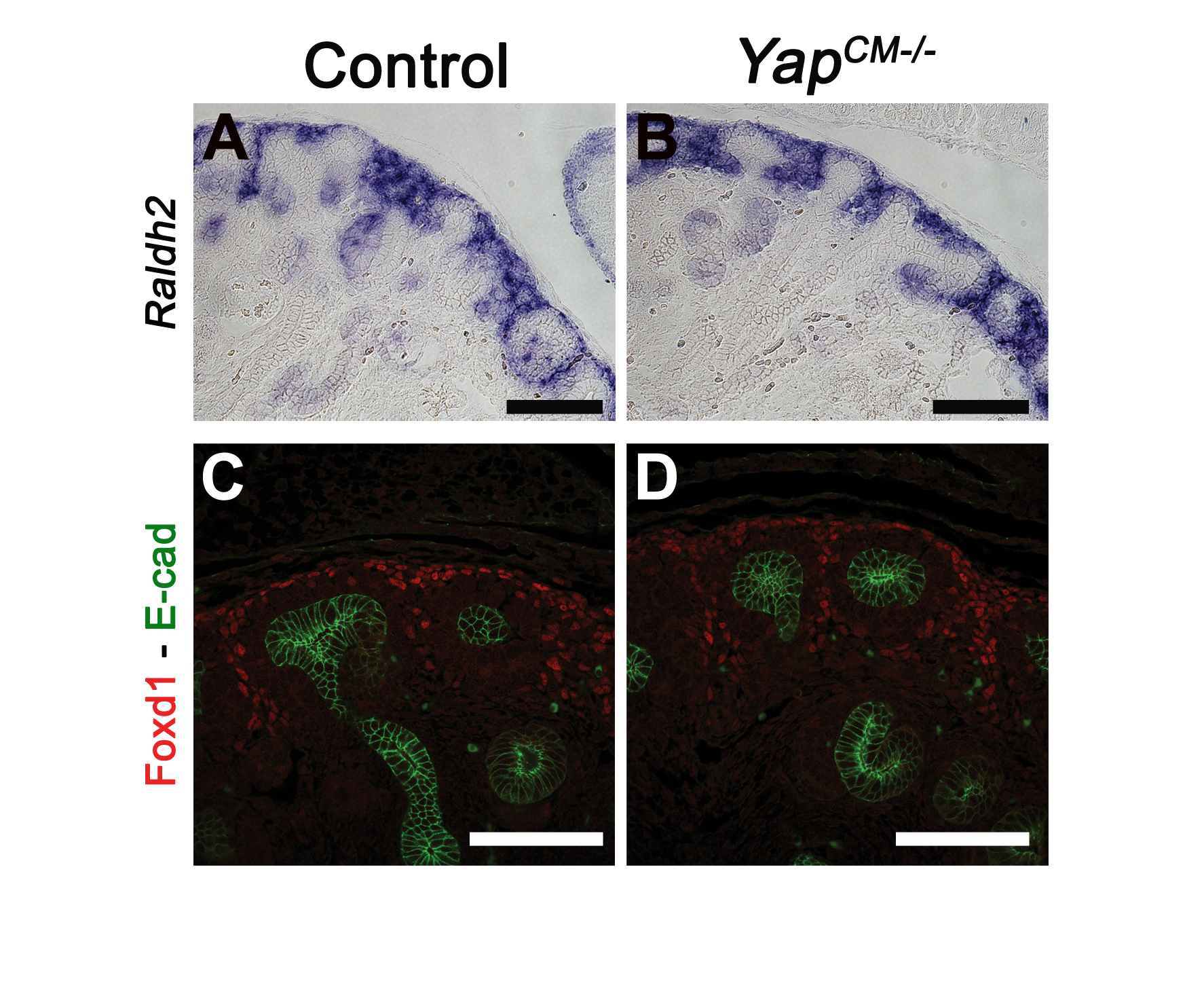

Supplement: Figure S13 — No change in stromal markers gene expression in Yap mutant. (A,B) ISH analysis reveals similar expression pattern of Raldh2 in both genotype. (C,D) Antibody staining for Foxd1 shows similar expression in both genotypes. E-cadherin was used to visualize the UB compartment. Scale bars represent 100 µm. (TIF) [file pgen.1003380.s013.tif]

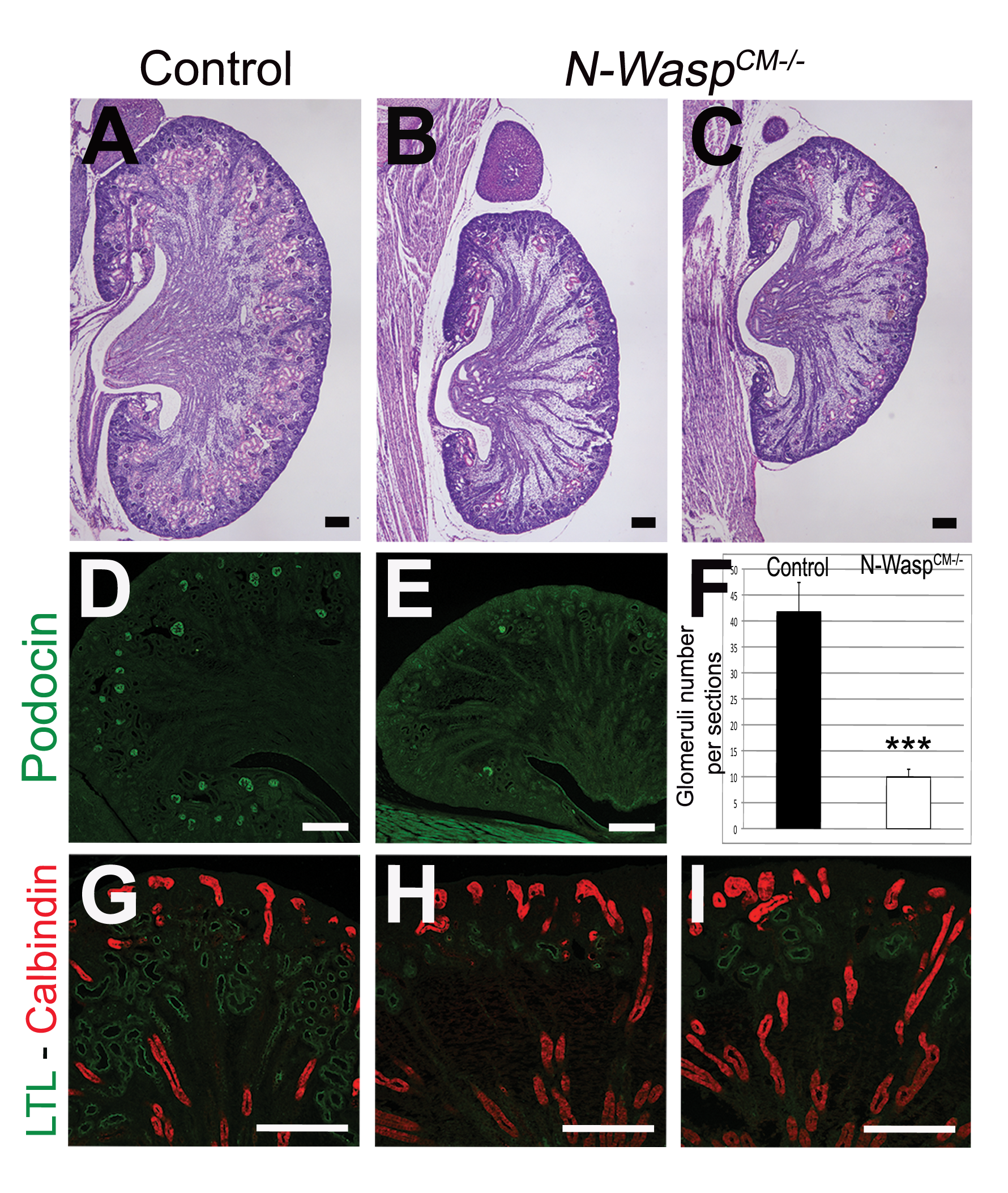

Supplement: Figure S14 — Loss of N-Wasp leads to hypoplasia and loss of glomeruli and proximal tubules. (A–C) PAS staining (P0) of wild-type and N-WaspCM−/− kidneys showing hypoplasia in mutants. (D,E) Sections of P0 kidneys processed for Podocin analysis shows a strong decrease in glomeruli formation in N-WaspCM−/− mutant kidneys. Average count and standard deviation from four controls and four mutants are shown in F. ***p<0.0001. (G–I) Sections of P0 kidneys processed for LTL and Calbindin staining shows reduced proximal tubule formation in N-WaspCM−/− mutant kidneys. Scale bars represent 100 µm (A–E), 200 µm (G–I). (TIF) [file pgen.1003380.s014.tif]

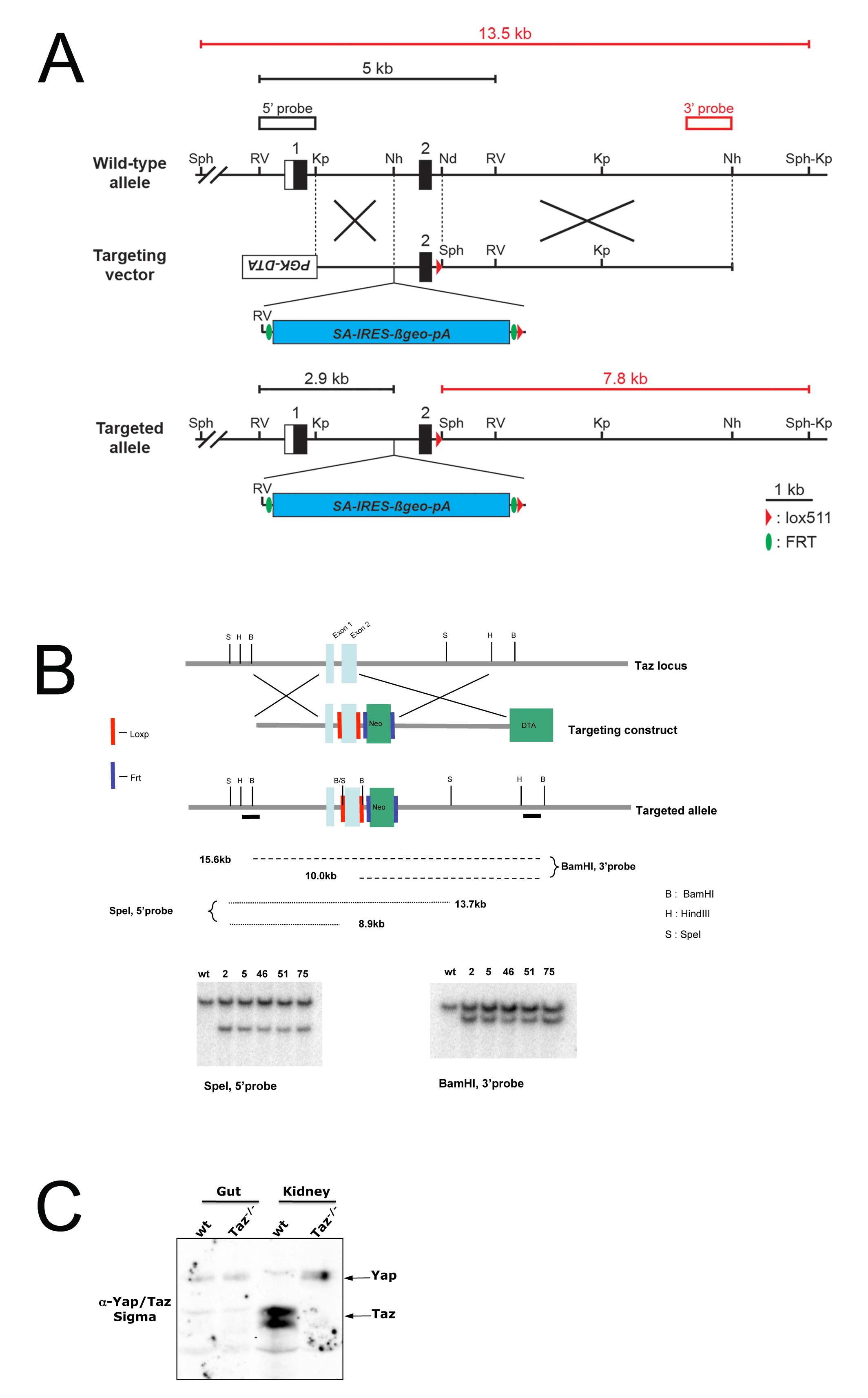

Supplement: Figure S15 — Generation of Yap and Taz flox allele. (A) Yap flox allele was generated by inserting LoxP sites for Cre-mediated excision flanking exons 2. (B) The Taz flox allele was generated by inserting LoxP sites for Cre-mediated excision flanking exons 2. (C) Western-blot analysis using Taz antibodies reveals absence of Taz protein in the Taz−/− kidneys. (TIF) [file pgen.1003380.s015.tif]
