# Supplementary material for: Yap- and Cdc42-Dependent Nephrogenesis and Morphogenesis during Mouse Kidney Development
Source: PLoS Genet. 2013 Mar 21;9(3):e1003380. doi: 10.1371/journal.pgen.1003380 (PMC3605093; doi:10.1371/journal.pgen.1003380)
Supplement: Table S1 — Candidates genes of microarrays on E13.5 YapCM−/− mutant kidneys compared to Yapflox/+ controls. All 24 genes were assayed by ISH and/or antibody staining on E14.5 wild-type and YapCM−/− kidneys. (PDF) [file pgen.1003380.s016.pdf]

Table S1

Symbol	Name	Fold change	Confirmed
**Upregulated**			
Actb	Actin, beta	2.833	N
Ret	Ret proto-oncogene	1.779	Y
Raldh3	Retinaldehyde dehydrogenase 3	1.581	Y
Fgf10	Fibroblast growth factor 10	1.566	Y
**Downregulated**			
Ly6a	Lymphocyte antigen 6 complex, locus A	-2.303	N
Meox2	Mesenchyme homeobox 2	-2.128	Y
Pdzk1	PDZ domain containing 1	-2.039	N
Sostdc1	Sclerostin domain containing 1	-1.963	Y
Yap	Yes-associated protein	-1.813	Y
Pitrm1	Pitrilysin metallopeptidase 1	-1.755	N
Capn6	Calpain 6	-1.726	Y
Cited1	Cbp/p300-interacting transactivator with Glu/Asp-rich carboxy-terminal domain 1	-1.642	Y
Traf1	Tnf receptor-associated factor 1	-1.583	Y
Osr2	Odd-skipped related 2	-1.576	N
Uncx4.1	UNC homeobox	-1.571	Y
Hnf4α	Hepatic nuclear factor 4, alpha	-1.539	N
Irx2	Iroquois homeobox 2	-1.494	N
Fgf8	Fibroblast growth factor 8	-1.466	N
Sox9	Sry-related HMG-box, gene 9	-1.436	N
Hnf1β	Hepatic nuclear factor 1, beta	-1.392	N
Lhx1	LIM homeobox protein 1 (Lim1)	-1.376	N
Ncam1	Neural cell adhesion molecule 1	-1.336	Y
Pax8	Paired box gene 8	-1.314	N
Pax2	Paired box gene 2	-1.285	Y
